# Recent Advances in the Development of Metal-Glycoconjugates for Medicinal Applications

**DOI:** 10.3390/molecules30173537

**Published:** 2025-08-29

**Authors:** Federica Brescia, Ioannis Titilas, Simona Cacciapuoti, Luca Ronconi

**Affiliations:** School of Biological and Chemical Sciences, University of Galway, University Road, H91 TK33 Galway, Ireland; f.brescia1@universityofgalway.ie (F.B.); john351990@hotmail.com (I.T.); simona.cacciapuoti@universityofgalway.ie (S.C.)

**Keywords:** glycoconjugates, metallodrugs, tumor metabolism, Warburg effect, targeted anticancer chemotherapy, chemotherapeutics, tumor imaging, antivirals, antimicrobials

## Abstract

Rapidly proliferating tumor cells exhibit elevated demands for nutrients and energy to support their uncontrolled growth, with glucose serving as a key metabolic substrate. Glucose is transported into cells *via* facilitated diffusion mediated by glucose transporters (GLUTs), after which it undergoes a series of enzymatic reactions to generate energy. To accommodate their heightened metabolic needs, cancer cells frequently overexpress GLUTs, thereby enhancing glucose uptake. Notably, aerobic glycolysis—commonly referred to as the “Warburg effect”—has been identified as the predominant pathway of glucose metabolism within tumor tissues, even in the presence of adequate oxygen levels. Consequently, the conjugation of chemotherapeutic agents, including metallodrugs, to glucose-mimicking substrates holds significant potential for achieving tumor-specific intracellular drug delivery by exploiting the elevated glucose uptake characteristic of cancer cells. Moreover, in recent years, glycosylation of metal scaffolds has been extended to the development of bioactive metallodrugs for applications other than cancer treatment, such as potential tumor imaging, antiviral, antimicrobial, antiparasitic and anti-neurodegenerative agents. Accordingly, major advancements in the design of metal-based glycoconjugates for medicinal applications are here summarized and critically discussed, focusing on related results and discoveries published subsequently to our previous (2015) review article on the topic.

## 1. Introduction

Over the last 60 years, metal complexes have increasingly taken on a central role in medicinal chemistry, demonstrating unique properties, ranging from the ability to bind DNA and modulate metabolic pathways to the controlled production of reactive oxygen species (ROS), for therapeutic and diagnostic applications [1]. Thanks to their varied coordination geometries, accessibility to multiple oxidation states, and tunable physico-chemical (including photophysical) features, metal ions allow for the design of molecules with high specificity for biological targets, multi-level mechanisms of action, and pharmacokinetic profiles that are unlikely to be achieved with “purely organic” species. In particular, the integration of appropriately chosen ligands allows the modulation of lipophilicity, tissue selectivity, and release kinetics, thus providing a rational optimization opportunity that has led to the FDA approval of some metal-containing drugs and the advancement of several new candidates into clinical trials [2].

**Figure 1:**
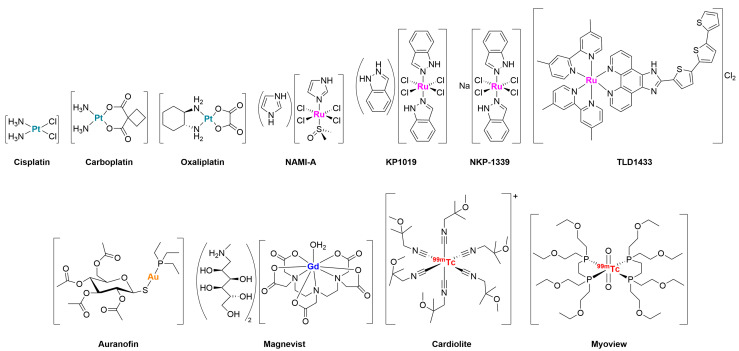
Representative examples of metallodrugs in clinical use or currently undergoing advanced clinical trials.

Among the metal complexes in clinical use and/or currently undergoing clinical trials (Figure 1), platinum derivatives represent the most well-known and widely employed class of drugs in oncology. Cisplatin, first introduced in 1978, is still largely used today for the treatment of testicular, ovarian, and bladder cancers due to its ability to bind to DNA and form cytoplasmic adducts with glutathione and metallothioneins, leading to the disruption of various cellular processes, which ultimately result in cell death [3]. Subsequently, carboplatin (1989) and oxaliplatin (2002) were developed, the latter being particularly effective in the treatment of colorectal carcinomas and showing reduced toxicity and the capability to partially overcome resistance mechanisms compared with cisplatin [4]. The clinical success of these compounds has reinforced the importance of platinum in chemotherapy but also highlighted its limitations due to renal toxicity, peripheral neuropathy, and the development of resistance to treatment [5].

The experience gained with platinum-based metallodrugs has stimulated research into other metals that could offer complementary advantages. For example, ruthenium, with its +2/+3 oxidation states, has been the subject of numerous (pre)clinical studies. In particular, NAMI-A and KP1019 (and its successor NKP-1339) have reached Phase I/II clinical trials for the treatment of metastatic solid tumors and leukemia, combining a tolerable toxicity profile with an antimetastatic action mediated by mechanisms that are not strictly cytotoxic [6,7,8]. More recently, the Ru(II) complex TLD1433 has passed Phase I for phototherapy of bladder carcinoma, exploiting the ability of ruthenium to generate ROS upon excitation with visible light, a process known as photodynamic therapy (PDT) [9]. Some gold derivatives, such as Auranofin, approved in 1985 for the treatment of rheumatoid arthritis, are now being reconsidered as potential anticancer agents. Their preferential inhibition of the selenium-containing enzyme thioredoxin reductase (TrxR), which is responsible for multiple intracellular processes including DNA synthesis, transcriptional regulation, cell growth, and resistance to oxidative agents, inducing oxidative stress and apoptosis, has been shown to effectively block the proliferation of hematological tumor cell lines and to sensitize chemoresistant cells. Auranofin is also undergoing clinical trials for the treatment of infectious diseases, such as the intestinal infection giardiasis (clinical trial ID NCT02736968), HIV (NCT02961829), and tuberculosis (NCT02968927) [10].

Metal complexes have been proving especially successful in diagnostic applications. Gadolinium derivatives in particular are widely used in magnetic resonance imaging (MRI). For instance, the contrast agent Magnevist, a diethylenetriaminepentaacetic acid (DTPA) derivative of gadolinium marketed since 1988, has been revolutionizing the diagnostic sector in oncology and neurology through the exploitation of the paramagnetic properties of the Gd(III) center to generate high-resolution MRI scans [11]. The continuous effort to increase the stability of the Gd(III) ion has led to extensive focus on the development of gadolinium-based nanoparticles in a view to overcoming the limitations of the traditional contrast agents, reducing the risk of nephrogenic systemic fibrosis and allowing faster clearance [12]. Similarly, the metastable element technetium-99m is an ideal radioisotope for single photon emission computed tomography (SPECT) [13] and, upon conjugation to ligands such as sestamibi or tetrofosmin led to the drugs Cardiolite and Myoview, respectively, used for myocardial perfusion and oncology diagnostics due to the emission of γ photons at 140 keV which enables the acquisition of high resolution images with relatively low radiation doses [14].

In addition to metallodrugs already approved or in advanced stages of clinical development, several complexes based on other metals, *e.g.,* iridium and rhenium, are being explored for therapeutic and diagnostic applications [2,15,16].

The clinical success and ongoing progress on metal-based chemotherapeutic and tumor diagnostic agents indeed support the potential of this class of small molecules to remain at the forefront of pharmaceutical research in the coming years. However, major challenges have to be considered, especially when it comes to optimizing selectivity towards diseased tissues/sites, fully understanding resistance mechanisms, improving biodistribution, and reducing potential adverse effects related to metal accumulation in healthy tissues. One promising way to tackle such issues is the rational design of metal complexes targeting specific features that differentiate tumor tissues from their healthy counterparts.

Unlike “normal” (*i.e.,* healthy) cells, neoplastic cells—whether benign or malignant—exhibit uncontrolled growth and replication, resulting in a continuous demand for nutrients and energy to support their aberrant proliferative activity [17]. Consequently, tumor cells undergo extensive metabolic reprogramming intended to provide a competitive advantage over adjacent stromal and immune cells, thereby facilitating access to the resources required for survival in an adverse microenvironment [18]. Amongst the several signature features of cancer discovered so far [19], targeting the major changes in glucose metabolism in cancer cells turns out to be a promising strategy in a view to developing targeted chemotherapeutics and diagnostics [20]. In this context, the functionalization of metal-containing scaffolds with carbohydrates may provide advantageous biological and physicochemical properties, such as increased water solubility, enantiomeric purity, stability, bioavailability, and reduced or even no toxicity. A particularly relevant aspect is the potential to achieve tumor selectivity due to the presence of sugar-like ligands, which can exploit upregulated signaling pathways and/or glycolytic enzymes active in the altered metabolism of tumor cells [21,22].

Within this framework, the present review paper aims at providing a comprehensive overview of the advances made over the past—broadly speaking—decade in the field of metal-glycoconjugates and their potential biomedical applications as (targeted) chemotherapeutic and tumor-imaging agents, focusing on small-molecule systems that employ various metal centers, including platinum, gold, ruthenium, copper, and others. While metal-glycoconjugation spans a broad and rapidly evolving field, this review specifically excludes topics such as cyclodextrin derivatives, nanoparticle-based platforms, polymeric carriers, and macromolecular assemblies, which are considered beyond its scope. Instead, the last section also discusses new research trends in the field beyond oncological applications, such as the development of metal-glycoconjugates as antiviral, antimicrobial, antiparasitic and anti-neurodegenerative agents.

## 2. Why Targeting Tumor Glucose Metabolism?

### 2.1. The “Warburg Effect” and Its Consequences

The rapid proliferation of malignant cells places extraordinary demands on nutrient (especially glucose) uptake and metabolic reprogramming. Unlike their normal counterparts, which rely primarily on mitochondrial oxidative phosphorylation (OXPHOS) to generate the energy needed for their sustenance from glucose, cancer cells preferentially rely on aerobic glycolysis, an observation first reported by Otto Warburg in the 1920s and now universally known as the “Warburg effect” [23,24]. In addition, the limited vascularization often found in solid tumors creates hypoxic microenvironments, which further promote glycolysis as the primary route for adenosine-5’-triphosphate (ATP) generation in cancer [25].

Under oxygen-rich conditions, normal cells process glucose through glycolysis, breaking it down into two molecules of pyruvate while producing two ATP molecules. Pyruvate is then transported into the mitochondria, where it is converted into acetyl-coenzyme A (acetyl-CoA) by the pyruvate dehydrogenase complex. Acetyl-CoA enters the Krebs cycle and is fully oxidized to two molecules of CO_2_, while electrons are transferred to generate three nicotinamide adenine dinucleotide (NADH) molecules and one flavin adenine dinucleotide (FADH_2_) molecule. These electron carrier molecules then donate their electrons to the mitochondrial respiratory chain, where their reoxidation drives the production of ATP from ADP and inorganic phosphate. Through this OXPHOS process, approximately 30 ATP molecules can be synthesized per glucose molecule taken up, compared to only 2 ATPs produced during glycolysis alone [26].

**Figure 2:**
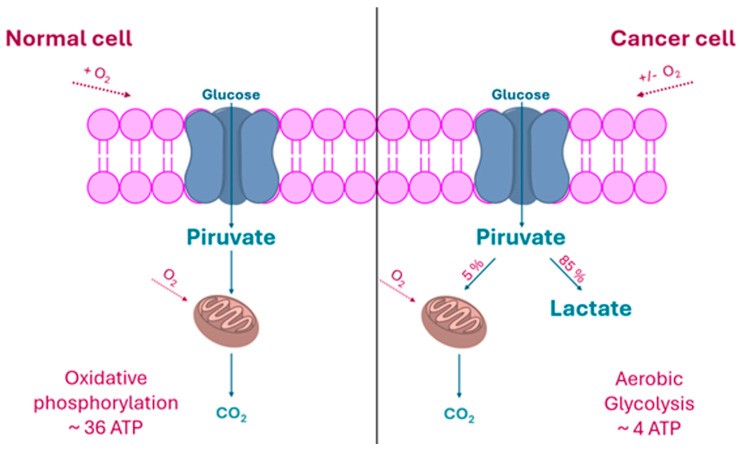
Simplified schematic representation of the glucose metabolic pathways, oxidative phosphorylation (OXPHOS) and aerobic glycolysis.

Oxygen plays a key role in glucose metabolism, acting as the final electron acceptor and allowing the full oxidation of glucose. In conditions where oxygen is depleted, such as in skeletal muscle during intense activity, cells shift to anaerobic metabolism and rely mostly on glycolysis, which is a much less efficient but faster process for the instant generation of energy. Under these circumstances, pyruvate is reduced to lactate through lactic fermentation, regenerating NAD^+^ from NADH to sustain the glycolytic flux [27]. A schematic overview of these distinct metabolic pathways is shown in Figure 2.

The upregulation in tumors of multiple genes involved in glycolysis, including lactate dehydrogenase, supports the idea that cancer cells exhibit an enhanced glycolytic flux [28]. This observation aligns with previous studies on cancer metabolism, which have shown that cancer cells preferentially rely on aerobic glycolysis to meet their energy demands even in the presence of oxygen, leading to elevated lactate production and its accumulation in the extracellular environment—consistent with the increased acidity of tumor sites [29,30,31,32]. At the molecular level, in cancer cells, proto-oncogenes like c-Myc become upregulated and signaling cascades such as the PI3K/Akt pathway are modified to support survival and growth. A central player in this adaptation is the transcription factor hypoxia-inducible factor 1 alpha (HIF-1α), which enhances transcription of the enzymes directly involved in the aerobic glycolysis [30,31]. Specifically, HIF-1α activates the transcription of genes coding for glucose transporters (GLUTs) and key glycolytic enzymes, thereby boosting the cell’s capacity to take up and metabolize glucose through glycolysis. It also upregulates pyruvate dehydrogenase kinase 1 (PDK1), an enzyme that inhibits the conversion of pyruvate into acetyl-CoA, effectively reducing the mitochondrial OXPHOS process. In parallel, HIF-1α promotes mitochondrial autophagy (mitophagy), helping eliminate mitochondria that might otherwise contribute to excessive production of ROS under oxidative stress [33]. In practice, this altered metabolism allows cancer cells to shunt glycolytic intermediates into anabolic pathways, producing nicotinamide adenine dinucleotide phosphate (NADPH), lipids, nucleotides, and non-essential amino acids, while excreting excess carbon as lactate [34]. In essence, aerobic glycolysis supports rapid biomass accumulation at the expense of efficient ATP production, enabling tumor cells to proliferate in environments where nutrient supply can be limited or highly variable [18].

### 2.2. The Rationale of Metal-Carbohydrate Conjugation

The carbohydrate-metal conjugation approach is based on a solid rational strategy for oncological targeting. Carbohydrates are transported into cells *via* GLUTs, a class of membrane proteins that facilitate the translocation of sugars (particularly glucose) as well as glyco-mimetic compounds across the cellular membrane [35]. Fourteen GLUTs (GLUT1-14) have been discovered to date, each exhibiting selective affinity for and recognition of specific monosaccharides [36]. Many tumors are known to overexpress GLUTs—in particular GLUT1 but in some cases also GLUT3, GLUT5, or sodium-dependent transporters (SGLTs)—to support their higher metabolic demand of glucose. Therefore, incorporating a sugar (*e.g.,* glucose, 2-deoxyglucose, galactose, mannose, glucosamine) into the formulation of a bioactive metal complex would make the overall metal-glycoconjugate recognizable by, and possibly preferentially internalized through, such transporters overexpressed in tumor cells [37]. A pioneering example is the radiopharmaceutical [^18^F]-2-fluoro-2-deoxy-d-glucose used as a positron emission tomography (PET) radiotracer: once absorbed, it is phosphorylated and trapped in the cell, allowing for the mapping of metabolically active tumor areas [38]. Applying this concept to metallodrug design would lead to the preferential accumulation in tumors, thus resulting in increased intracellular concentration of the drug precisely where it is needed.

A second important aspect is that the incorporation of sugar moieties is expected to significantly increase the water solubility of metal complexes, thus improving their pharmacokinetic properties [39]. An additional level of selectivity may be achieved with prodrugs of metal-glycoconjugates designed in such a way as to activate only within or in proximity of the tumor microenvironment. In this case, the sugar moiety can be removed by enzymes present in the tumor (*e.g.,* β-glucuronidase in the antibody-directed enzyme prodrug therapy (ADEPT)) or under conditions typical of tumors, such as hypoxia or acidic pH, triggering the activation of the drug only at the diseased site [22,40].

A major boost to research in the field was provided by the resolution of the crystal structures of human GLUT1 and its bacterial homologs (such as XylE), which have revealed that the hydroxyl groups at positions C^1^ through C^5^ of glucose form essential hydrogen bonds with polar residues in the binding pocket (*i.e.,* Gln282, Asn288, Asn317) critical for substrate recognition. In contrast, the hydroxyl group at position C^6^ is oriented outwards and does not seem to participate in these key interactions [41,42,43]. These structural insights have made the C^6^ position the preferred site for chemical modifications, including metal coordination, as it preserves the transporter’s binding affinity [44].

Glucose remains the most obvious and widely used sugar moiety. However, analogues such as 2-deoxy-d-glucose (2-DG) and 2-fluoro-2-deoxy-d-glucose (2-FDG) provide a “dual advantage”: they are efficiently internalized inside the cell *via* GLUTs, and are known to subsequently interfere with glycolysis since they lack the C^2^-OH group and, therefore, they cannot undergo isomerization to fructose-6-phosphate, an essential step in aerobic glycolysis [45].

Galactose and mannose, although less common as substrates for GLUTs, may offer greater specificity for certain isoforms such as GLUT3 over GLUT1 [46,47]. Additionally, derivatives such as glucosamine and glucuronic acid allow for the rational design of prodrugs that are selectively activated by tumor-associated enzymes (*e.g.,* β-glucuronidase) [48]. The anomeric configuration (α or β) at the C^1^ position can also significantly influence biological activity. While both forms may retain similar affinity for GLUTs, α-anomers have been shown in some cases to exhibit greater biological efficacy, highlighting the importance of evaluating both configurations during the design of glycoconjugates [49].

To summarize, metal-carbohydrate conjugation enables the integration of multiple pharmacological advantages within a single molecular architecture:

- Metabolic targeting *via* GLUTs overexpressed in tumors.
- Improved pharmacokinetic properties (*e.g.,* water solubility, biodistribution).
- Reduced systemic toxicity.
- Possibility of controlled activation within the tumor microenvironment through enzyme-mediated cleavage.

These features make metal-glycoconjugates highly promising candidates for the development of selective therapeutic agents in oncology and beyond.

## 3. Metal-Glycoconjugates for Oncological Applications

### 3.1. Platinum Complexes

#### 3.1.1. Platinum(II)-Glycoconjugates

As previously mentioned, Pt(II) complexes have long held a central role in chemotherapy, with drugs such as cisplatin, carboplatin, and oxaliplatin being at the forefront of cancer treatment. Their chemotherapeutic activity relies primarily on their ability to form covalent adducts with DNA, especially at the N^7^ sites of neighboring guanines, forming (mostly) intra-strand cross-links that block replication and transcription, ultimately triggering apoptosis [50].

In an effort to improve both selectivity and efficacy, a number of recent studies have explored the functionalization of Pt(II) pharmacophores with sugar moieties, giving rise to a growing class of Pt(II)-glycoconjugates. These constructs are often designed to exploit the increased expression of GLUTs (particularly GLUT1) in many tumors, thereby promoting preferential uptake into cancer cells while reducing systemic toxicity and increasing water solubility to improve pharmacokinetic properties [51].

In this section, we will first discuss glycoconjugates that retain the classical oxaliplatin scaffold, where the carbohydrate is linked to modulate pharmacokinetics and uptake without drastically altering the core geometry of the metal complex. We will then move on to compounds featuring entirely different coordination environments and ligand architectures, including non-classical Pt(II) complexes with trigonal bipyramidal (TBP) geometries. Notably, while many of these conjugates were designed to preserve the canonical DNA binding mechanism of cisplatin derivatives, others display alternative or additional modes of action, including interaction with cellular proteins, redox modulation, or mitochondrial targeting. These mechanistic variations may offer new avenues for overcoming resistance and improving therapeutic outcomes.

**Figure 3:**
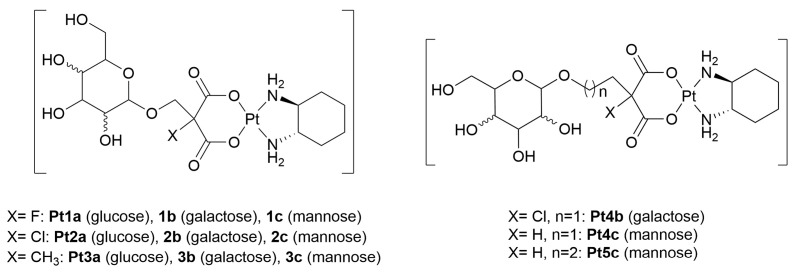
Chemical structures of some representative Pt(II)-glycoconjugates bearing an oxaliplatin-like core developed by Gao and co-workers [52,55,56,57].

Gao and co-workers designed a series of Pt(II)-glycoconjugates in which glucose, galactose, or mannose residues were linked to the oxalato scaffold of oxaliplatin (Figure 3). Compounds **Pt1–2x** were tested against six human tumor cell lines, including lung carcinoma (A549), colorectal adenocarcinoma (HT-29), melanoma (A-375), epidermoid carcinoma (A-431), esophageal carcinoma (Eca-109), and ovarian adenocarcinoma (SK-OV-3) [52]. All metal-glycoconjugates exhibited superior cytotoxicity (1.5- to 6-fold) compared to oxaliplatin, particularly in GLUT1-overexpressing lines such as A-431 [53]. The improved uptake was attributed to GLUT1-mediated transport, as demonstrated by fluorescent competition assays using the GLUT1 probe 2-NBDG (2-[*N*-(7-nitrobenz-2-oxa-1,3-diazol-4-yl)amino]-2-deoxy-d-glucose), and further confirmed through inhibition studies with quercetin, a known GLUT1 blocker [54]. Molecular docking simulations revealed that the sugar motifs of these conjugates bind at the same site as endogenous sugars on GLUT1, supporting their proposed mechanism of selective transport into cancer cells.

This study was extended to the 2-methylmalonato analogues **Pt3x** [55], achieving remarkable improvements in water solubility, especially for the glucose derivative, which showed a 530-fold increase compared to cisplatin. These conjugates maintained or exceeded the cytotoxicity of oxaliplatin across the same panel of cancer cell lines and showed a strong dependence on GLUT-mediated uptake, as demonstrated by both competition assays with 2-NBDG and functional inhibition with quercetin. DNA binding studies confirmed that the new compounds formed guanine adducts as efficiently as, or better than, oxaliplatin, with the galactose conjugate showing the fastest kinetics. Notably, *in vivo* evaluation in a murine leukemia model (L1210) revealed a significantly improved safety profile for **Pt3a**, with higher maximum tolerated doses (MTD), reduced systemic toxicity, and improved survival rates compared to both cisplatin and oxaliplatin.

Building upon such positive results, the same research group further developed this class of complexes to capitalize on GLUT-mediated uptake for the treatment of non-small cell lung cancer and colorectal cancer [56]. The galactose-conjugated Pt(II) derivative **Pt4b** in particular exhibited improved water solubility and pronounced cytotoxicity in GLUT1-overexpressing cell lines such as lung carcinoma H460 cells [58]. Quercetin inhibition studies again confirmed GLUT-dependent uptake, and fluorescence competition assays validated **Pt4b** as a competitive substrate for GLUT1. The complex also displayed *in vivo* a markedly higher therapeutic index than oxaliplatin and cisplatin, with reduced toxicity and superior tumor inhibition in mouse models of lung and colon cancer, despite being administered at lower relative doses.

In a complementary study [57], Gao *et al.* reported on two novel mannose-conjugated Pt(II) complexes (**Pt4c** and **Pt5c**) exhibiting drastically improved aqueous solubility, up to 100-fold higher solubility than oxaliplatin. Both compounds demonstrated potent cytotoxicity across a range of cancer cell lines, with particularly strong activity against GLUT1-rich HT-29 cancer cells. Functional assays revealed that activity was strongly dependent on GLUT1 expression: cytotoxicity was attenuated in GLUT1-silenced cells and enhanced in GLUT1-overexpressing cells. Uptake quantification revealed an eightfold increase in intracellular platinum in GLUT1-positive conditions. Mechanistic investigations confirmed these complexes induced apoptosis through mitochondrial pathways, as evidenced by caspase-3 activation, suppression of anti-apoptotic markers (Bcl-2, Bcl-XL), and DNA fragmentation through terminal deoxynucleotidyl transferase dUTP nick-end labeling (TUNEL) assay. Remarkably, *in vivo* studies showed for **Pt4c** a threefold higher MTD compared to oxaliplatin and a significantly extended survival rate in mice bearing L1210 leukemia, without inducing observable toxicity.

**Figure 4:**
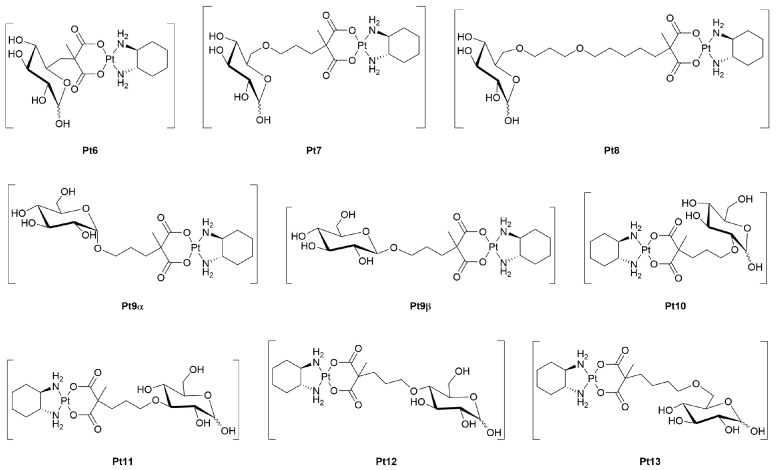
Chemical structures of some representative Pt(II)-glycoconjugates bearing an oxaliplatin-like core developed by Lippard and co-workers [44,49].

Lippard and co-workers provided further insights into the field of Pt(II)-glycoconjugates by exploring in detail the effect of glucose-conjugation of oxaliplatin (Figure 4), which emphasized the role of GLUT-mediated uptake for achieving selective anticancer effects. The authors generated three glucose-functionalized oxaliplatin derivatives (**Pt6–8**) [44], differing in the length of the spacer linking the glucose moiety and the Pt(II) pharmacophore. These compounds were rationally designed based on the structural features of glucose transporters, particularly focusing on modifications at the C^6^ position of glucose to preserve recognition by GLUT1. Among the series, **Pt6** turned out as the most promising, showing superior uptake and cytotoxicity across a panel of cancer cell lines, including ovarian adenocarcinoma A2780 cells, where IC_50_ values reached sub-micromolar levels (0.15–0.22 μM). Uptake inhibition studies confirmed the involvement of GLUT1: the presence of known GLUT1 inhibitors such as phloretin or 4,6-*O*-ethylidene-α-d-glucose (EDG), or high concentrations of d-glucose, reduced intracellular accumulation of **Pt6** by approximately 50%. Notably, l-glucose had no effect, supporting the transporter-specific mechanism of uptake. The study also revealed a secondary contribution of the Organic Cation Transporter 2 (OCT2) to the cellular internalization of both **Pt6** and oxaliplatin, as demonstrated by competition assays using cimetidine [59]. Selectivity was rigorously evaluated using matched pairs of normal and cancerous epithelial cells derived from prostate and kidney tissues. **Pt6** exhibited markedly higher accumulation and cytotoxicity in cancer cells, in line with the overexpression of GLUT1 and OCT2, while sparing normal cells. Moreover, despite GLUT1 expression in neuronal cells [60], **Pt6** displayed significantly reduced potency in this context, suggesting a favorable therapeutic window and reduced risk of neurotoxicity compared to traditional platinum drugs.

In a subsequent study [49], the authors undertook a comprehensive structure–activity investigation by synthesizing all positional isomers of a glucose-oxaliplatin complex, in which the Pt(II) scaffold was attached to various sites of the glucose ring (**Pt9–13**). This allowed a thorough investigation of the influence of sugar conjugation site on GLUT1-mediated uptake, cytotoxicity, and selectivity. Among the isomers, conjugation to the C^1^α and C^2^ positions proved the most effective, with the latter glycoconjugate (**Pt10**) returning the highest cell uptake and cytotoxicity across multiple cancer cell lines. Mechanistic studies confirmed that the cytotoxic activity correlated with GLUT1 expression since in GLUT1-knockdown prostate carcinoma DU145 cells, uptake and cytotoxicity of **Pt10** significantly diminished. The order of GLUT1 specificity followed the site conjugation trend C^2^ (**Pt10**) > C^1^α (**Pt9α**) ≈ C^1^β (**Pt9β**) > C^3^ (**Pt11**) ≈ C^4^ (**Pt12**) ≈ C^6^ (**Pt13**), indicating that both the position and anomeric configuration of the glycosidic linkage are crucial for efficient GLUT-mediated uptake. Importantly, in matched cancer *vs.* normal cells, all isomers displayed minimal uptake in normal cells, again pointing to GLUT1 as a key determinant of selectivity. **Pt10** was further evaluated for its anticancer activity *in vivo* using a mouse model of breast cancer overexpressing GLUT1. The compound was shown to accumulate preferentially in the tumor tissue over normal organs and showed significant antitumor activity, reinforcing the translational potential of rationally designed Pt(II)-glucose conjugates for targeted chemotherapy. Altogether, these studies provided compelling evidence that the conjugation of a sugar moiety to Pt(II) complexes may be advantageous in terms of selectivity toward cancer cells. This improved selectivity not only increases the likelihood of effective tumor targeting but also contributes to a more favorable toxicity profile, potentially reducing the burden of side effects commonly associated with conventional platinum-based therapies.

**Figure 5:**
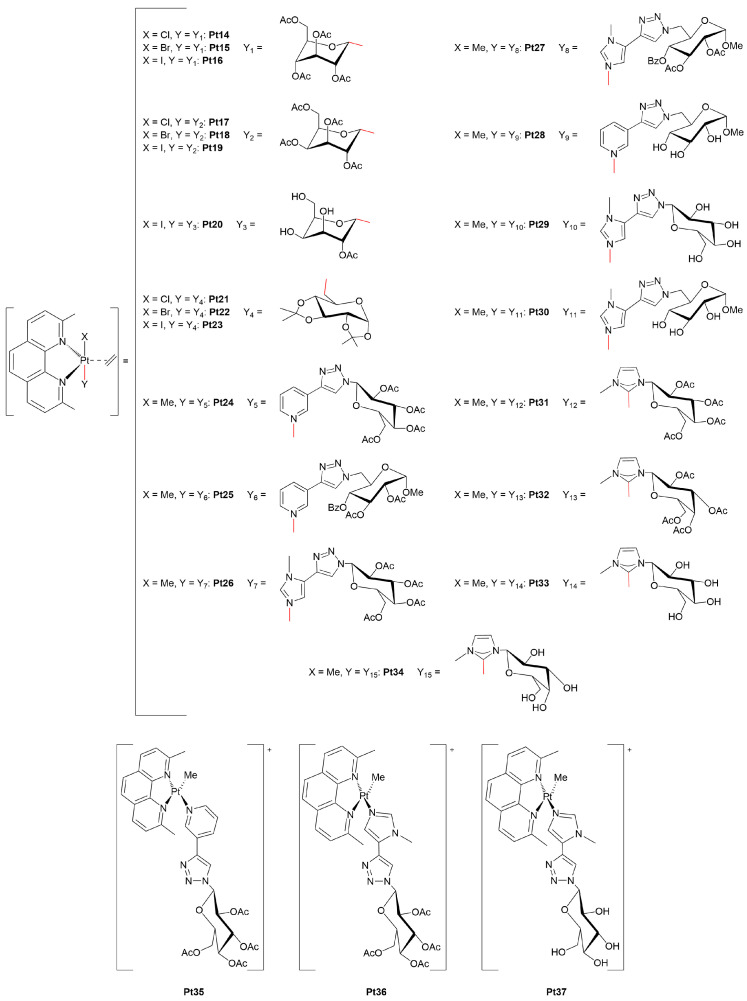
Chemical structures of some representative Pt(II)-glycoconjugates developed by Ruffo and co-workers [61,62,63,64].

With an interest in expanding the structural diversity of Pt(II)-glycoconjugates, Ruffo and co-workers explored the coordination chemistry and stability of Pt(II) complexes showing unusual TBP geometry (Figure 5). The authors developed a series of C-glycosylated Pt(II) derivatives, in which galactose or glucose moieties were directly bonded to the metal center via carbon atoms at either the C^1^ or C^6^ position [61] The resulting complexes exhibited a TBP geometry, with the sugar unit and an halide or methyl residue occupying the axial positions, while the equatorial plane was defined by a bidentate nitrogen ligand and an ethene molecule. Biological evaluation on matched immortalized non-tumorigenic (BALB/c-3T3) and SV40 virus-transformed (SV-T2) murine fibroblast cell lines showed that galactose-bearing complexes, especially **Pt17** and **Pt19**, exerted significantly higher cytotoxicity towards cancer cells. For instance, **Pt17** had an IC_50_ of 3.9 μg mL^−1^ (≅4.8 μM) in SV-T2 cells compared to 15.2 μg mL^−1^ (≅18.7 μM) in normal fibroblasts, while cisplatin exhibited no such selectivity. Interestingly, glucose derivatives did not have the same selectivity profile, highlighting the importance of the type of sugar within the same structural framework. Mechanistic studies supported a GLUT1-mediated uptake, as evidenced by the decreased cytotoxicity upon quercetin treatment, while deacetylation of the sugar removed tumor selectivity and increased general toxicity. Furthermore, the galactose complexes were shown to induce apoptosis *via* mitochondrial and oxidative stress pathways, and also displayed strong DNA binding affinity, particularly for the iodo derivatives.

In a related contribution [62], the authors extended the structural diversity of this class of Pt(II)-glycoconjugates by introducing triazole-linked sugars using a Cu(I)-catalyzed azide–alkyne cycloaddition (CuAAC), also known as click-chemistry. The resulting complexes maintained the same TBP geometry through a chelating *N*,*N*′-ligand and ethene, but featured sugar moieties attached *via* triazolylpyridine or triazolylimidazole linkers. *In vitro* studies revealed potent cytotoxicity across both cancer (A-431 and breast adenocarcinoma MCF-7) and non-cancerous (myoblast H9c2 and immortalized human keratinocyte HaCaT) cell lines, significantly outperforming cisplatin in terms of absolute potency, although exhibiting limited tumor selectivity. Beyond cellular assays, the authors also investigated interactions with biomacromolecules. Ethidium bromide displacement and circular dichroism experiments confirmed that these complexes bind DNA, leading to perturbations of base stacking and helicity. Furthermore, a high-resolution X-ray crystal structure proved that one of the triazole-Pt(II) complexes (**Pt26**) formed a stable adduct with bovine pancreatic ribonuclease (RNase) A upon coordination of the His105 side-chain while preserving the five-coordinate geometry and ethene ligand, which was the first crystallographic evidence of a five-coordinate platinum-protein complex. Remarkably, the enzymatic activity of RNase A was not inhibited, indicating a non-destructive interaction with the protein scaffold.

Building upon these findings, Ruffo and collaborators further advanced the development of Pt(II)-glycoconjugates by systematically exploring the biological properties and mechanism(s) of action of five-coordinate Pt(II) complexes. In their 2021 study [63], the authors examined a structurally diverse set of metal-glycoconjugates, some C- and N-linked glycosylated complexes previously reported [61,62], and others newly designed *N*-heterocyclic carbene (NHC) Pt(II) species, to assess how different sugar-metal linkages would impact tumor selectivity, stability, and cytotoxic profiles. The C-bonded complexes (**Pt14–23**) retained their TBP geometry and exhibited selective cytotoxicity towards cancer cells. For example, compound **Pt19**, featuring a galactose ligand bound at the C^1^ position, showed an IC_50_ of 4.5 μM in tumor cells *vs.* 21 μM in normal fibroblasts. These values clearly surpassed those of cisplatin, which remained broadly cytotoxic with minimal selectivity. Functional studies confirmed that this selectivity could be partly attributed to GLUT1-mediated uptake, as quercetin significantly reduced their overall cytotoxicity, in line with previous evidence of glucose transporter engagement. In contrast, N-linked glycoconjugates (**Pt24–30**), bearing sugar units attached *via* glycosylated pyridine or imidazole, showed substantially lower selectivity, possibly due to the partial loss or lability of the sugar moiety in aqueous solution.

The carbene complexes (**Pt31–34**), where the sugar moiety was integrated into an NHC framework, recorded extraordinary cytotoxicity and selectivity. In particular, compound **Pt31** showed an IC_50_ nearly 100 times lower and a selectivity index over 180 times higher than cisplatin. Intriguingly, the mechanism of action of this complex diverged from classical platinum drugs: rather than forming adducts with DNA, it modified DNA conformation, as observed through CD, and altered DNA topology in electrophoretic assays. These results pointed toward a non-classical mode of DNA interaction, possibly involving groove binding or external electrostatic interactions rather than direct DNA platination. Further studies confirmed the induction of apoptosis through mitochondrial disruption and ROS generation, supporting a multi-target cytotoxic mechanism.

In a follow-up study [64], the structure-activity relationship of Pt(II)-glycoconjugates was further investigated by directly comparing square-planar and TBP Pt(II) analogues all incorporating triazole-based glucose ligands. The goal was to determine how the coordination environment affects GLUT1 targeting, cellular uptake, and biological selectivity. The tetracoordinated square-planar complexes **Pt 35–37** exhibited lower overall cytotoxicity but markedly improved selectivity toward cancer cells compared to their TBP counterparts (**Pt24,26,29**). Among those, the pyridine-containing complex **Pt35** showed a selectivity index significantly higher than both cisplatin and the corresponding pentacoordinated complex. Importantly, GLUT1 inhibition had no effect on cytotoxicity, indicating that, despite the presence of glucose moieties, their uptake was not relying on GLUT-mediated transport, as discussed in the previous article. Mechanistically, the square-planar complexes induced mitochondria-mediated apoptosis without causing necrosis, as evidenced by caspase-3 and -9 activation and the disruption of mitochondrial membrane potential in A-431 cancer cells. DNA binding assays suggested that these complexes interacted primarily through non-covalent intercalation, rather than covalent platination of DNA, and that sugar acetylation played a key role since unprotected derivatives, such as **Pt37**, showed minimal activity.

Overall, such detailed and extensive studies provided in-depth mechanistic insights into the understanding of how molecular geometry, carbohydrate anchoring strategy, and linker design can shape the biological performance of Pt(II)-glycoconjugates. In particular, they undoubtedly demonstrated that the rational diversification of the coordination environment can be successfully used to modulate solubility, stability, cellular behavior and biological targets.

#### 3.1.2. Platinum(IV)-Glycoconjugates

Beyond the extensively studied Pt(II)-glycoconjugates, Pt(IV) analogues have emerged as a distinct class of anticancer agents with unique structural and pharmacokinetic advantages [65]. Pt(IV) complexes may be regarded as pro-drugs designed to be reduced intracellularly to their Pt(II) counterparts, subsequently releasing cytotoxic species alongside the axial ligands. The nature of these ligands can strongly influence the pharmacological behavior, biodistribution, and mechanistic profile of the resulting complexes [66]. In this context, the conjugation of carbohydrate moieties to Pt(IV) scaffolds represents a promising strategy to refine both targeting and drug release properties. The following studies illustrate diverse approaches to the design of Pt(IV)-glycoconjugates and their impact on biological activity and selectivity.

**Figure 6:**
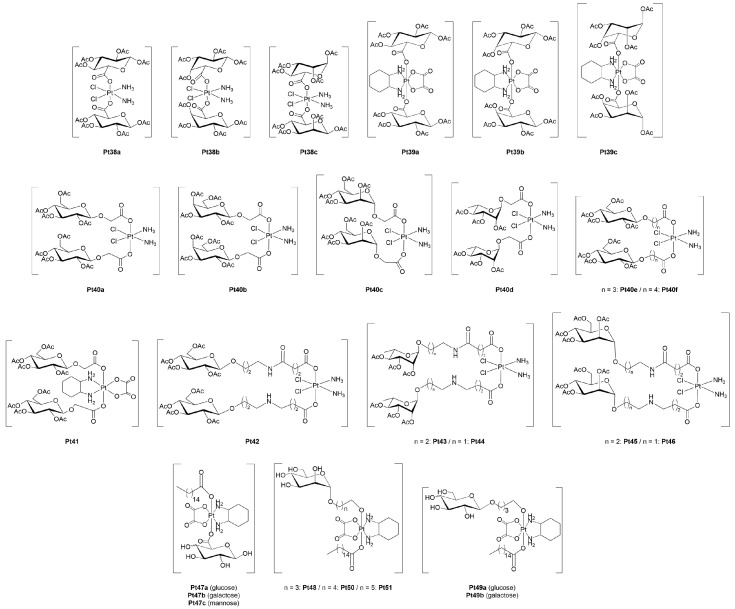
Chemical structures of some representative Pt(IV)-glycoconjugates developed by Wang and co-workers [67,68,69].

In a systematic body of work, Wang and co-workers explored a wide range of glycosylated Pt(IV) complexes, focusing primarily on the axial functionalization of cisplatin- and oxaliplatin-derived scaffolds with carbohydrate units (Figure 6). These studies illustrated the impact of the type of sugar, linker chemistry, and cellular context on cytotoxicity, selectivity, and uptake mechanisms.

In their first contribution [67], they reported the design, synthesis, and biological evaluation of a novel series of glycosylated Pt(IV) complexes, incorporating various monosaccharides (*i.e.,* glucose, galactose, mannose, rhamnose, and glucuronic acids), into Pt(IV) scaffolds derived from cisplatin and oxaliplatin (**Pt38–41**). All compounds were screened *in vitro* against a panel of six human tumor cell lines: cervical adenocarcinoma (HeLa), liver carcinoma (HepG2), prostate carcinoma (LNCap), MCF-7, A549 and the corresponding cisplatin-resistant A549R cells. Among the library, compounds **Pt38b**, **Pt39b** and **Pt40c** stood out as the most promising based on their superior cytotoxic profiles, and, as such, were selected for further mechanistic investigations. They proved to induce apoptosis in HepG2 cells and exhibited higher intracellular platinum accumulation and increased DNA binding compared to control complexes, suggesting improved internalization and target engagement. Remarkably, they also showed to retain significant cytotoxicity towards both A549 and A549R cells, highlighting their capability to overcome drug resistance by circumventing classical resistance mechanisms. Unexpectedly, cell uptake and cytotoxicity were not reduced upon inhibiting GLUT1, indicating that GLUT1 is likely not involved in the cellular internalization of these glycosylated Pt(IV) pro-drugs. Finally, the two most cytotoxic compounds, **Pt39b** and **Pt40c**, were tested in a HepG2 xenograft mouse model. Both significantly inhibited tumor growth *in vivo* and showed much lower systemic and organ toxicity than cisplatin, as evidenced by stable body weight throughout the treatment period.

In a subsequent work [68], the same authors presented the synthesis, structural characterization, and biological evaluation of a new series of glycosylated Pt(IV) pro-drugs (**Pt42–46**) based on a Pt(IV)-succinate precursor. The aim was to assess how variations in the sugar moiety and linker architecture would influence cytotoxicity, selectivity, and activation mechanisms. The glycoconjugates incorporated glucose, rhamnose, or mannose units, attached *via* either propyl amino or ethyl amino linkers. The most striking outcome of this study was the superior performance of the mannose-containing pro-drugs, especially against HeLa and LNCaP cancer cells. **Pt45** recorded IC_50_ values of 2.81 and 4.82 μM in HeLa and LNCaP cells, respectively, accounting for a 2- to 5-fold increase in potency relative to cisplatin and oxaliplatin. Even more impressively, **Pt46** achieved an IC_50_ of 1.90 μM in LNCaP cells, making it the most cytotoxic compound of the series. Importantly, both compounds exhibited high selectivity for cancer cells over normal fibroblasts (3T3), with IC_50_ values of 84 μM (**Pt45**) and 169 μM (**Pt46**) against the latter. These values indicate a wide therapeutic window and reduced systemic toxicity compared to classical Pt(II) drugs. The difference in performance between the two complexes underscored the critical influence of the length and flexibility of the linker, with the shorter chain in **Pt46** inducing greater potency and selectivity. Mechanistic studies confirmed that the mannose-based glycoconjugates accumulated more efficiently in cancer cells, leading to significantly higher platinum uptake and DNA platination than cisplatin or oxaliplatin. The authors also assessed the redox activation potential of the pro-drugs by reacting with ascorbic acid, mimicking the reducing environment of tumors. Upon reduction, the Pt(IV) complexes were converted into the corresponding active Pt(II) species capable of forming bifunctional DNA adducts. *In vivo* data further confirmed the translational promise of the mannose conjugates. In fact, both **Pt45** and **Pt46** showed MTD and LD_50_ nearly four times higher than oxaliplatin, indicating low systemic toxicity.

Amongst the various articles authored by Wang and co-workers, it is worth citing a comprehensive research paper in which they evaluated a family of eight glycosylated Pt(IV) complexes (**Pt47–51**) incorporating different carbohydrates (glucose, galactose, mannose, glucuronic acid), various linker lengths and attachment positions (C^1^ or C^6^), and in some cases, a long-chain fatty acid (hexadecanoic acid) designed to enable binding to human serum albumin (HSA) [69]. The glycosylated complexes displayed superior antitumor potency, with IC_50_ values as low as 0.19 μM, far surpassing standard clinical drugs like cisplatin, oxaliplatin, and, in some instances, even satraplatin, by up to 166-fold. Importantly, the compounds also showed significantly reduced cytotoxicity in non-cancerous cells such as kidney 293T cells and 3T3 fibroblasts, underscoring their high therapeutic selectivity. Among the series, the C^1^-glucose-substituted compound **Pt49a** emerged as the lead candidate, showing the best combination of cytotoxic potency, GLUT-mediated uptake, and tumor selectivity. Mechanistic studies demonstrated that several of these compounds, including **Pt47a**, **Pt47c** and **Pt49a**, could effectively kill cisplatin- and oxaliplatin-resistant cancer cells, highlighting their potential to overcome chemoresistance. Uptake experiments, including co-treatment with EDG, phloretin and cimetidine, confirmed that such glycosylated Pt(IV) complexes function as substrates for both GLUT1 and OCT2 transporters. The C^1^-linked derivatives were particularly efficient in exploiting those transporters, whereas the C^6^-bound analogues showed limited capability to target GLUTs. Beyond cellular uptake, the introduction of hexadecanoic acid chains conferred strong non-covalent binding to HSA, markedly improving drug stability in the bloodstream and extending circulation time. *In vitro*, several complexes exhibited half-lives exceeding 170 h, significantly outperforming Pt(II) drugs. Albumin binding also had the effect of slowing ascorbate-mediated reduction, contributing to better tumor accumulation and delayed activation until intracellular delivery was achieved. The *in vivo* efficacy was evaluated in a breast cancer xenograft mouse model, where compound **Pt49a** proved once again to be the most effective. It achieved over sixfold higher accumulation in tumor tissues compared to controls, leading to a major inhibition of tumor growth, and induced minimal body weight loss, indicating low systemic toxicity. Such studies demonstrated that this class of Pt(IV)-glycoconjugates acts as dual substrates for GLUTs and OCTs, while also exploiting HSA-mediated delivery. These findings also suggest a future direction toward orally available formulations, owing to the high chemical stability of the pro-drugs.

**Figure 7:**
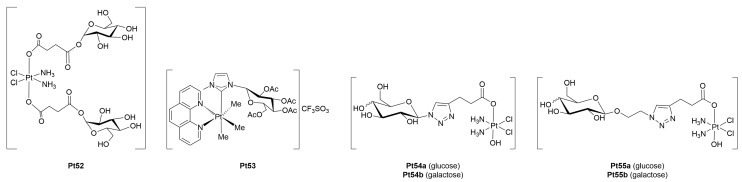
Chemical structures of some representative platinum(IV)-glycoconjugates developed by Wu [70], Ruffo [71], Montagner [72] and co-workers.

The potential advantages of glycosylated Pt(IV) pro-drugs in improving tumor selectivity and overcoming the limitations of Pt(II)-based chemotherapeutics have been recently explored by other research groups (Figure 7).

Wu *et al.* [70] developed a Pt(IV)-glucoconjugate pro-drug (**Pt52**) by chemically modifying cisplatin to incorporate a glucose moiety. Biological evaluations, carried out across several cell lines, including gastric cancer (SGC7901), their glucose transport-deficient variant (SGC-7901R), HepG2, HeLa cells, and normal murine fibroblasts (L-929), showed significantly enhanced uptake of the complex in GLUT-overexpressing cancer cells compared with unmodified cisplatin, supporting the involvement of GLUTs in its internalization. Conversely, in the SGC7901R line characterized by impaired glucose transport, uptake, and cytotoxicity dropped markedly, an effect not observed with cisplatin, whose cell entry mostly occurs by passive diffusion and is non-selective. Importantly, HepG2 cells, known for high GLUTs expression [73], showed the greatest accumulation and sensitivity to **Pt52**, with a clear correlation between intracellular platinum levels and cell death induction. Meanwhile, normal L929 fibroblasts demonstrated low uptake and high viability when treated with the complex, indicating reduced off-target toxicity.

Ruffo *et al.* extended their research on platinum-glycoconjugates by providing an insightful comparative study between the glycosylated platinum(II) complex **Pt31** (Figure 5) and its Pt(IV) analogue **Pt53** [71]. From a chemical standpoint, the latter was stable in DMSO but underwent hydrolysis in aqueous medium, leading to the detachment of the sugar moiety and the consequent disruption of both targeting and stability features. Conversely, the Pt(II) complex maintained integrity in water but was prone to ligand exchange in organic solvents. Interestingly, both showed improved stability when incubated with DNA or proteins, suggesting a potential stabilizing mechanism *in vivo*. **Pt31** exhibited remarkable cytotoxicity *in vitro*, up to 100-fold greater than cisplatin, and high selectivity for cancer cells, while its Pt(IV) counterpart was largely inactive, showing poor selectivity and weak cytotoxic effects even at high concentrations. Importantly, **Pt53** and its hydrolysis product(s) were found to be extremely resistant to chemical reduction by common intracellular reductants such as ascorbate and glutathione. Electrochemical analyses supported this finding, revealing highly negative reduction potentials that explain the lack of intracellular activation, which is a requirement for Pt(IV) pro-drugs to exert cytotoxic effects. Mechanistic studies confirmed that both **Pt31** and **Pt-53** can bind DNA, forming well-defined 1:1 adducts with DNA duplexes.

Building on the diverse body of evidence supporting the therapeutic value of Pt(IV)-glycoconjugates, Montagner *et al.* [72] contributed to the field a distinct and disease-specific perspective by targeting osteosarcoma, a highly aggressive malignancy of the bone predominantly affecting young patients [74]. Four novel Pt(IV)-carbohydrate complexes (**Pt54–55x**) were generated and tested. Biological evaluation began with 2D cell culture assays, where all complexes demonstrated dose-dependent cytotoxicity against MG-63 and SAOS-2 osteosarcoma cell lines, outperforming cisplatin at equivalent concentrations. Just as importantly, the compounds exhibited markedly reduced toxicity toward healthy osteoblasts (hFOBs), highlighting their improved therapeutic window. This selective activity was attributed to preferential uptake in cancer cells, consistent with the overexpression of GLUTs in osteosarcoma. Studies performed in a 3D bone-mimetic scaffold model seeded with MG-63 cells showed that all complexes, and in particular those bearing galactose moieties, again surpassed cisplatin in suppressing tumor cell viability. These findings not only consolidate the anticancer potential of these Pt(IV) pro-drugs but also point to a possibly underappreciated role of galactose metabolism in osteosarcoma biology, opening new avenues for therapeutic targeting.

#### 3.1.3. Are Platinum(0)-Glycoconjugates a Feasible Alternative?

As previously discussed, both the (even) minimal structural variations of the coordination environment and the oxidation state of the metal center influence the bioactivity of platinum-based glycoconjugates. In this context, it is equally valuable to consider a less explored oxidation state: Pt(0).

**Figure 8:**
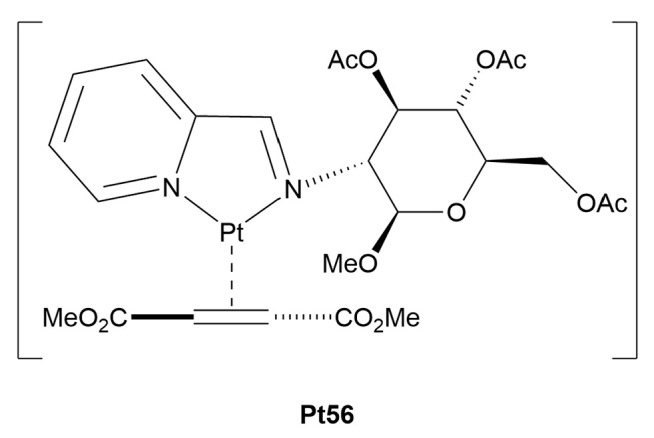
Chemical structure of the Pt(0)-glycoconjugate developed by Ruffo [75] and co-workers.

The study reported by Ruffo and co-workers [75], to the best of our knowledge, represents the first report of a water-soluble Pt(0) complex bearing a glucoconjugated ligand, not an easy challenge, if we consider the scarcity of information relating to aqueous organometallic chemistry of Group 10 elements in low oxidation states [76,77]. The authors synthesized a novel Pt(0) complex, **Pt56** (Figure 8), where a bidentate glucoconjugated 2-iminopyridine is coordinated to platinum alongside dimethylfumarate. The resulting complex is highly soluble in both organic and aqueous media and is remarkably stable, maintaining its structure for over a week in solution. This stability, along with the absence of reactivity toward oxidative addition, underscores its potential applications under physiological conditions, where premature decomposition is a common limitation for many metal-based drugs. The anticancer activity of the complex was evaluated against four cell lines, showing moderate cytotoxicity across all cell types. Notably, in BALB/c-3T3 cells and SVT2 fibroblasts, it was more active than cisplatin, which had much higher IC_50_ values. However, unlike other glycoconjugated Pt(II) complexes, this Pt(0) derivative lacked clear selectivity between cancerous and non-cancerous cells, suggesting that additional structural refinements may be necessary to achieve tumor-specific targeting.

### 3.2. Ruthenium Complexes

#### 3.2.1. Ruthenium(II)-Glycoconjugates

**Figure 9:**
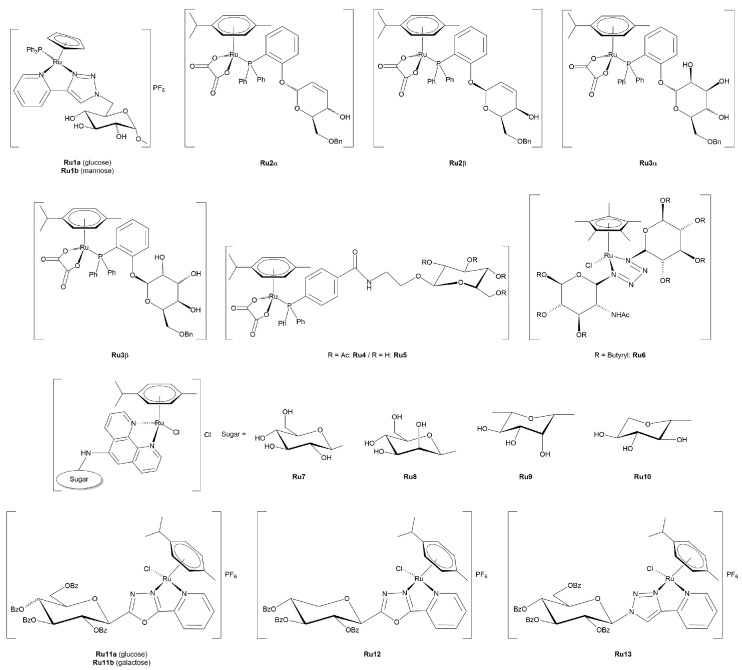
Chemical structures of some representative Ru(II)-glycoconjugates developed by Fernandes [83], Di Bussolo [84], Pinkas [85], Royo [86], Bokor [87] and co-workers.

Ru(II) complexes have emerged as promising alternatives to platinum-based chemotherapeutics, offering distinct advantages such as low systemic toxicity, activity against cisplatin-resistant cancers, and selectivity for cancer cells, often through the exploitation of overexpressed transferrin receptors [78,79]. Moreover, Ru(II) compounds can mimic iron in biological systems, favoring interaction with biomolecules beyond DNA [80]. In particular, cyclopentadienyl (Cp)-Ru(II) complexes have shown potent cytotoxicity across various cancer cell lines [81]. The prototype compound [Ru(η^5^-C_5_H_5_)(bipy)(PPh_3_)](PF_6_) inhibits enzyme poly(ADP-ribose) polymerase 1 (PARP-1) and induces apoptosis, with several functionalized derivatives exhibiting improved efficacy or altered cellular behavior [82]. In this context, the glycoconjugation of Ru(II) scaffolds, especially for the so-called half-sandwich architectures such as cyclopentadienyl- or arene-Ru(II) complexes, has gained increasing attention as a strategy to improve solubility, selectivity, and cellular uptake *via* GLUTs. The studies discussed in this section all relate to the generation and biological activity of half-sandwich Ru(II) scaffolds variously linked to carbohydrate units (Figure 9).

A representative example of this approach is provided by the work of Fernandes and co-workers [83], who designed a series of Cp-Ru(II) complexes of the type [(η^5^-C_5_H_5_)Ru(N^N)(PPh_3_)](PF_6_), including two carbohydrate-appended derivatives: **Ru1a** (glucose) and **Ru1b** (mannose). While those metal-glycoconjugates exhibited slightly lower cytotoxicity against colorectal carcinoma (HCT116) cells, their mechanism of action revealed a distinct advantage. Through a competition assay with d- and l-glucose, the authors demonstrated that their cytotoxicity was significantly attenuated in the presence of excess d-glucose, but unaffected by l-glucose. This strongly supports GLUT-mediated uptake, and computational docking studies further validated this mechanism, showing that the glucose moiety of these complexes fits the glucose-binding pocket of the XylE transporter homolog. To the best of our knowledge, these findings make complexes **Ru1a** and **Ru1b** as the first ruthenium-based anticancer agents proven to enter cells *via* GLUTs, emphasizing the potential of carbohydrate conjugation not only to modulate solubility and selectivity but also to enhance active targeting through cancer-specific metabolic pathways.

A complementary approach to glycosylated Ru(II) complexes was presented by Di Bussolo and collaborators [84], who developed a series of *p*-cymene-Ru(II) half-sandwich complexes functionalized with novel glycophosphane ligands (**Ru2–5**). These ligands were designed to incorporate a variety of carbohydrate fragments, including rare unsaturated sugars such as d-galactal, directly into the phosphane moiety. Among the compounds tested, the 2,3-unsaturated glycoside derivatives **Ru2α** and **Ru2β** displayed potent cytotoxicity across multiple cancer cell lines, including cisplatin-resistant A2780R cells. Notably, **Ru2β** consistently outperformed both its α-anomer (**Ru2α**) and cisplatin, with IC_50_ values in the low micromolar range (3–7 μM), and showed rapid and efficient cellular uptake. Mechanistic studies revealed that **Ru2β** caused G2/M and S phase cell cycle arrest, induced apoptosis *via* caspase-3 and -7 activation, and disrupted mitochondrial membrane potential in over 80% of cells, suggesting a mechanism of action distinct from DNA-targeting drugs like cisplatin. Despite their potency, these complexes showed limited selectivity between cancerous and normal cell lines, highlighting a key challenge for future optimization. Nevertheless, this work demonstrates that stereochemistry and glycoside structure critically influence activity and pave the way to further explore glycoconjugation *via* phosphane ligands as a robust method for engineering Ru(II)-based metallodrugs.

An alternative strategy to ruthenium(II) glycoconjugation was reported by Pinkas and collaborators [85], who developed a series of tetrazene-based Cp-Ru(II) half-sandwich complexes functionalized with various glucose-like moieties. The goal was to evaluate how sugar identity and protection affect cytotoxicity and physicochemical properties. The most active compound (**Ru6**) exhibited substantial cytotoxicity, outperforming cisplatin by up to five-fold in both cisplatin-sensitive and -resistant ovarian (A2780, SK-OV-3) and triple-negative breast (MDA-MB-231) cancer cell lines. Interestingly, activity was strongly dependent on sugar protection: unprotected glycoconjugates were largely inactive, while acylated derivatives (*i.e.,* acetyl, propionyl, butyryl) recorded IC_50_ values in the micromolar range, correlating with an optimal degree of lipophilicity. Despite its potency, **Ru6** lacked tumor selectivity, affecting both cancerous and non-cancerous cells, and its mechanism of action was limited to apoptosis induction, with no clear effects on cell cycle or migration.

While most glycosylated Ru(II) complexes have been developed to enhance cytotoxicity or cancer cell selectivity, Royo *et al.* [86] explored a different therapeutic angle, that is, the modulation of metastasis-related processes. The authors designed a family of phenanthroline-based arene-Ru(II) half-sandwich complexes bearing different carbohydrate units (**Ru7–10**), aiming at evaluating how different sugar moieties would affect properties such as cell migration and matrix metalloproteinase (MMP) activity. Although all metal-glycoconjugates showed moderate cytotoxicity toward prostate adenocarcinoma (PC-3) cells, generally lower than the non-glycosylated analogue, the most promising results emerged from their anti-migratory and anti-MMP effects. All compounds significantly inhibited PC-3 cell migration in wound-healing assays and suppressed the activity of MMP-9, a key enzyme in extracellular matrix degradation during metastasis progression. Notably, the xylose-functionalized complex (**Ru10**) exhibited the strongest MMP-9 inhibition, while the mannose counterpart (**Ru8**) showed the strongest *in vitro* cytotoxic activity. Interestingly, GLUT inhibition did not affect cellular uptake, suggesting that these compounds do not rely on GLUTs for cellular internalization. These findings pointed towards a possible non-cytotoxic mechanism of action, paving the way for Ru(II)-based antimetastatic agents that impair cancer cell invasiveness rather than induce direct cell death.

To further explore alternative antitumor mechanisms, Bokor and co-workers developed new Ru(II) complexes featuring bidentate sugar-derived ligands, with a focus on triazole- and oxadiazole-linked monosaccharides with various protecting groups [87]. Amongst the several compounds generated, **Ru11–13** in particular showed strong cytostatic activity in ovarian cancer cell models (A2780 and ID8), with IC_50_ values as low as 0.87 μM, comparable or superior to cisplatin. Surprisingly, those complexes did not induce apoptosis or necrosis at the cytotoxic doses. Instead, they blocked cell proliferation by promoting lipid-associated oxidative stress. The effect was reversed by antioxidants like vitamin E, confirming ROS as the primary driver of activity. Remarkably, such effects were selective: the most active complex **Ru13** showed minimal toxicity toward normal fibroblasts, in contrast with the broader cytotoxicity observed for many metal-based drugs. Structure-activity analysis revealed that biological activity requires a protected sugar scaffold, with benzoyl groups being especially effective.

#### 3.2.2. Photoactivatable Ruthenium(II)-Glycoconjugates

**Figure 10:**
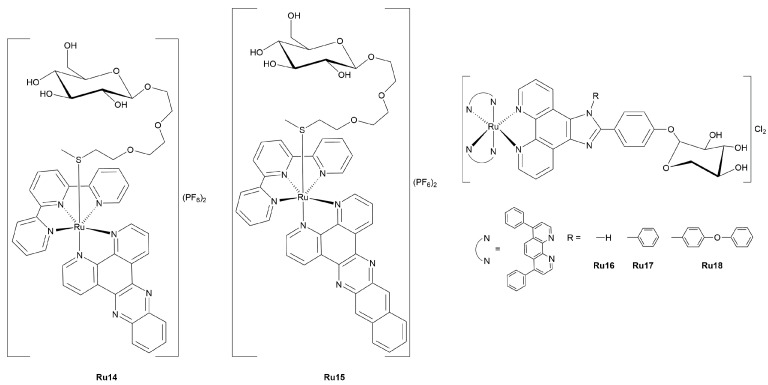
Chemical structures of some representative photoactivatable Ru(II)-glycoconjugates developed by Bonnet [89,90], Chao [91] and co-workers.

In recent years, Ru(II)-polypyridyl complexes have gained increasing attention as photoactivatable anticancer agents for PDT and photoactivated chemotherapy (PACT). These approaches aim to reduce systemic toxicity by activating the drug locally upon irradiation with light at the appropriate wavelength, leading to the generation of ROS or triggering cytotoxic effects in a controlled spatiotemporal manner [88]. To further improve selectivity and solubility, several studies have explored the conjugation of carbohydrate moieties to photoactive Ru(II) scaffolds (Figure 10).

Bonnet and co-workers developed a library of light-activatable polypyridyl-Ru(II) complexes conjugated to a glucose-like scaffold through a thioether linker [89]. Within this series, two complexes, namely **Ru14** and **Ru15**, stood out for their distinct photochemical and biological profiles. While the former exhibited the highest photosubstitution quantum yield (*i.e.,* the light-induced exchange of their thioether ligand for H_2_O), the latter proved to be the most effective in terms of biological activity. Upon blue light irradiation (at 450 nm), **Ru15** not only undergoes photosubstitution, ultimately releasing the cytotoxic Ru(II) species by cleaving the thioether-glucose ligand, but also acts as a potent photosensitizer, generating singlet oxygen (^1^O_2_) with high efficiency (*Φ*_Δ_ = 0.71). Such dual action results in strong photocytotoxicity (EC_50_ ≈ 0.7–0.8 µM in lung carcinoma (A549) and breast adenocarcinoma (MCF-7) cells), as well as negligible toxicity in the dark. The released photoproduct(s) would then accumulate in mitochondria, bind mitochondrial DNA, and contribute to cell death *via* combined oxidative and DNA-damaging mechanisms. Building on these promising results, the authors further investigated the mechanism of action of complex **Ru15** by synthesizing its l-glucose analogue, which, unlike d-glucose, is not expected to be recognized and subsequently internalized by GLUTs [90]. Unexpectedly, both isomers exhibited comparable mitochondrial localization and energy-independent cellular uptake, indicating that GLUTs’ involvement was negligible in this context. Although the d-glucose metal conjugate showed slightly higher dark toxicity, its phototoxicity was equivalent to that of the l-glucose analogue.

The range of PACT strategies was further expanded by Chao and co-workers, who developed a new class of Ru(II)-glucoconjugates specifically designed for two-photon PDT [91]. Recognizing the need to improve light penetration, mitochondrial targeting, and cancer selectivity, the authors synthesized four water-soluble complexes (**Ru16–18**) functionalized with d-glucose through the C^1^ position, preserving affinity for glucose transporters, and with bidentate *N*,*N*-ligands optimized for photophysical performance. These compounds combine selective uptake *via* GLUTs/SGLTs and preferential mitochondrial accumulation, with **Ru16** showing the most promising profile. Interestingly, the cellular uptake of **Ru16** decreased by almost 80% in the presence of either metabolic inhibitors or phlorizin (an SGLT inhibitor), suggesting that SGLTs were likely the main contributors to active transport. However, because neither treatment completely blocked uptake, approximately 20% of uptake appears to occur through energy-independent processes, likely involving GLUT-mediated or passive diffusion. **Ru16** exhibited the highest two-photon absorption cross-section (181 GM at 810 nm), excellent singlet oxygen generation, and strong photocytotoxicity upon two-photon irradiation, with negligible dark toxicity. Notably, *in vivo* studies in a HeLa xenograft mouse model showed complete inhibition of tumor growth after localized administration and irradiation, without body weight loss or systemic side effects. This dual-targeting strategy, ensuring selective uptake into cancer cells and preferential accumulation in mitochondria, represents a significant advance toward clinically viable two-photon-activated Ru(II)-glycoconjugates.

### 3.3. Gold Complexes

#### 3.3.1. Gold(I)-Glycoconjugates

**Figure 11:**
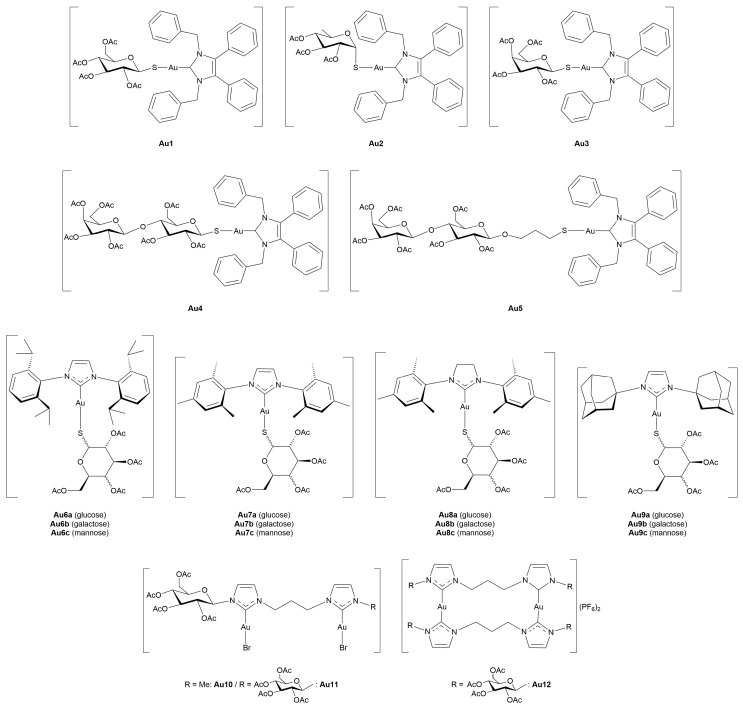
Chemical structures of some representative Au(I)-glycoconjugates developed by Zhu & Tacke [93], Nolan & Ott [94], Tubaro [95] and co-workers.

Au(I) complexes have been attracting growing interest in medicinal chemistry due to their unique linear coordination geometry, strong affinity for sulfur- and phosphorous-donor biomolecules, and promising anticancer properties [92]. When functionalized with carbohydrate moieties, these complexes can combine the pharmacological potential of gold with the advantageous biological features of sugars, such as water solubility, cell-specific recognition, and membrane transport modulation [21]. This section specifically focuses on NHC-Au(I)-glycoconjugates. Recent studies have advanced synthetic strategies to the generation of glycosylated Au(I) derivatives functionalized with mono- or disaccharide units, alongside the evaluation of their antiproliferative effects in various cancer cell lines (Figure 11). Altogether, these contributions highlight the versatility of Au(I) coordination chemistry in glyco-functional drug design and set the stage for more targeted, structure-based development of glycoconjugated metal drugs.

Tacke and collaborators reported on a series of NHC-Au(I) complexes functionalized with thiosugars as potential anticancer agents (**Au1–5**) [93]. Inspired by the Au(I) drug auranofin and its known capability to inhibit TrxR, the authors aimed at improving tumor selectivity and cellular uptake by incorporating carbohydrate units such as glucose (both α- and β-anomers), galactose, and lactose. All complexes displayed good water solubility, and their cytotoxicity was evaluated across the NCI-60 Human Tumor Cell Line Screening programme [96], returning promising IC_50_ values in the low micromolar range for most compounds. Notably, complex **Au2**, bearing an α-thioglucose moiety, exhibited the highest potency, reaching nanomolar activity against several tumor cell lines. Structure–activity relationship analysis highlighted the importance of the sugar unit, anomeric configuration, and the presence of a spacer group in modulating biological response.

A comprehensive study was reported by Nolan & Ott and co-workers, who designed a structurally diverse series of Au(I)-thiolato glycoconjugates bearing NHC ligands, introducing a simple, eco-friendly synthetic route with excellent yields and operational simplicity [94]. The authors employed a base-promoted ligand exchange reaction between [Au(NHC)Cl] precursors and thiosugar derivatives of glucose, galactose, and mannose, under mild experimental conditions. A streamlined one-pot protocol was also devised, allowing *in situ* generation of the NHC ligands from azolium salts. A total of twelve complexes (**Au6–9x**) featuring different *N*-heterocyclic carbene scaffolds—namely IPr (1,3-bis(2,6-diisopropylphenyl)imidazolidin-2-ylidene), IMes (1,3-bis(2,4,6-trimethylphenyl)imidazolidin-2-ylidene), SIMes (the saturated analogue of IMes), and IAd (1,3-bis(1-adamantyl)imidazolidin-2-ylidene)—were synthesized and characterized, with the structure of one complex (**Au9c**) confirmed by X-ray crystallography. *In vitro* cytotoxicity studies against A549, HT-29 and MDA-MB-231 human tumor cell lines revealed potent antiproliferative activity, with IC_50_ values in the sub-micromolar to low micromolar range. Notably, compounds bearing glucose residues proved more active, especially against breast cancer cells. This work demonstrates how a flexible, component-based design strategy combined with green chemistry principles can lead to highly biologically active Au(I)-glycoconjugates suitable for further drug development.

Tubaro *et al.* explored a different structural class of Au(I)-glycoconjugates by designing dinuclear complexes bearing bis-NHC ligands functionalized with acetylated glucopyranose moieties (**Au10–12**) [95]. The purpose of this study was to assess whether the incorporation of sugar moieties into multimetallic architectures could improve the anticancer potential of mono NHC-Au(I) systems previously synthesized. Biological evaluations showed that all dinuclear complexes displayed dose-dependent cytotoxicity, but with relatively high IC_50_ values (generally >100 μM), indicating only weak antiproliferative activity against the tested tumor cell lines (A-431, SV-T2). Functionalization with carbohydrate units had a limited impact on cytotoxicity, although the dicationic complex **Au12** exhibited slightly improved activity, likely due to enhanced mitochondrial accumulation driven by its lipophilic cationic nature. These results suggest that while carbohydrate groups can modulate solubility and charge distribution, glycosylation did not contribute to the biological efficacy in dinuclear Au(I) systems. The study nevertheless provides a valuable framework for future investigations into multimetallic glycoconjugates and supports the notion that charge and mitochondrial targeting may be more critical determinants of activity than sugar incorporation alone.

#### 3.3.2. Gold(III)-Glycoconjugates

Although Au(I) complexes have been widely investigated for their biological activity and chemical robustness, Au(III) compounds also hold significant therapeutic promise due to their structural similarities with Pt(II), such as sharing the same electron configuration and the tendency to achieve square-planar coordination geometries suitable, at least in principle, for nucleobase or protein binding [97]. However, the development of Au(III)-based drugs has been historically limited by their intrinsic instability in biological environments, where reduction to inactive Au(I) species—or even to metallic gold—often readily occurs [98]. To address this challenge, recent studies have focused on ligand frameworks that stabilize the Au(III) center while providing additional pharmacological benefits.

**Figure 12:**
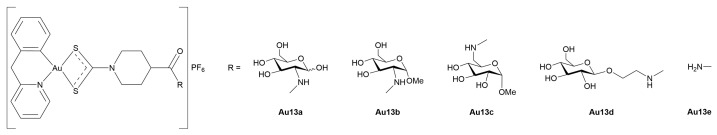
Chemical structures of some representative Au(III)-glycoconjugates developed by Ronconi [99] and co-workers.

A representative example of rationally designed Au(III)-glycoconjugates is provided by the work of Ronconi and collaborators, who reported on a library of organogold(III)-dithiocarbamato complexes functionalized with glucosamine-like precursor (**Au13x**, Figure 12) [99]. The study focused on evaluating how the position of glycosylation on the sugar scaffold influences both biological activity and cellular uptake, leveraging the differential recognition of GLUTs. Among the four Au(III)-glycoconjugates tested, complex **Au13c**, bearing a glucose moiety conjugated at the C^6^ position, proved the most promising candidate. In proliferation assays against the cisplatin-sensitive ovarian carcinoma A2780 cell line, the complex showed the lowest GI_50_ value (7.1 μM), significantly outperforming its analogues, which exhibited GI_50_ values ranging from 12.7 to 14.1 μM. The authors attributed this enhanced activity to the greater compatibility of C^6^-glycosylation with GLUT-mediated transport [41], compared to the more sterically hindered and conformationally constrained C^1^ or C^2^ conjugations. Surprisingly, the co-administration of a GLUT1 inhibitor (EDG) paradoxically increased the potency of **Au13c** (GI_50_ dropped to 3.3 μM), suggesting that cancer cells may compensate *via* upregulation of alternative glucose transporters, such as GLUT3, which might transport this compound more effectively. **Au13c** was also selected for mechanistic studies, alongside the best corresponding non-glycosylated analogue **Au13e**, and demonstrated a multi-targeted mode of action. Flow cytometry revealed G_2_/M cell cycle arrest and mitochondrial disruption, while biochemical assays confirmed the dual inhibition of topoisomerase I and II, as well as the increased generation of ROS. These effects, though less pronounced than those observed with non-glycosylated analogue **Au13e**, indicate that glycosylation does not abolish the core cytotoxic mechanism but may modulate its intensity and delivery. Furthermore, cellular accumulation studies highlighted a clear distinction in uptake mechanism: while the **Au13e** entered cells predominantly by passive diffusion, **Au13c** showed saturable, carrier-mediated transport, consistent with its hydrophilic, carbohydrate-decorated nature. Although the overall accumulation of **Au13c** was lower, this behavior suggests the potential for selective uptake by cancer cells exploiting glucose transport pathways. Taken together, these findings position **Au13c** as a promising lead compound within the field of Au(III)-glycoconjugates.

### 3.4. Miscellaneous Metal Complexes with Anticancer and Antimicrobial Activity

#### 3.4.1. Monometallic-Glycoconjugates

As previously mentioned, the potential of metal-glycoconjugates based on metal centers other than platinum has received growing attention only in recent years [100]. In several cases, researchers investigated how the exchange of the metal core within a conserved ligand framework would modulate biological properties, thus offering a direct comparison between different metal cores [101]. Those studies provide valuable insights into the influence of metal’s identity on cytotoxicity, stability, and cellular uptake when the overall architecture of the conjugate remains constant. Beyond such comparative approaches, other studies have ventured into the use of less conventional metals, including palladium, osmium, magnesium, iridium, iron, and zinc, in combination with sugar-containing ligands. These systems often diverge from classical coordination geometries and mechanisms of action, expanding the field of metal-based glycoconjugation.

**Figure 13:**
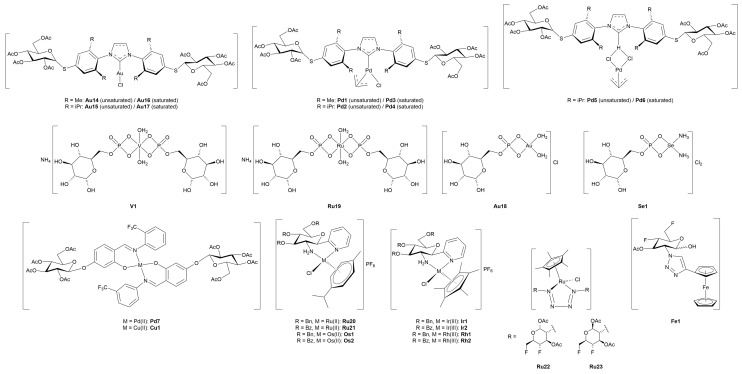
Chemical structures of some representative metal-glycoconjugates developed by Scattolin [102], Al-Wasidi [103], Morales [104], Bokor [105], Karban [106] and co-workers.

This section opens with studies that extend known metal-carbohydrate platforms into a direct metal exchange paradigm, where the same glycosylated ligand scaffold is preserved and the only variable is the metal center (Figure 13).

A representative example of this approach was provided by Scattolin and co-workers [102], who synthesized a series of *N*-heterocyclic carbene complexes functionalized with thioglucoside units and coordinating either Au(I) (**Au14–17**) or Pd(II) (**Pd1–6).** Starting from azolium salts bearing thioglucoside groups, the authors developed three families of compounds: Au(II)-NHC-thioglucosides, Nolan-type Pd(II)-allyl NHC complexes, and neutral allyl palladates. Among the gold complexes, **Au15** and especially **Au17**, which contain saturated NHC ligands, displayed the highest potency *in vitro*, with **Au17** outperforming cisplatin in A2780 cells and showing high activity against the more aggressive ovarian carcinoma OVCAR-5 cell line. In contrast, the Pd(II)-allyl complexes **Pd1** and **Pd2**, based on unsaturated NHC ligands, showed superior activity among the Pd(II) series, with the latter rivalling cisplatin’s efficacy not only in A2780 and OVCAR-5, but also maintaining activity in the cisplatin-resistant A2780cis cell line, suggesting a potentially distinct mechanism of action. Meanwhile, the neutral palladates **Pd5** and **Pd6** showed comparable cytotoxicity across the three cancer cell lines, regardless of the NHC saturation state. Crucially, most of the thioglucoside-bearing complexes exhibited minimal toxicity toward healthy fibroblasts (IC_50_ > 100 µM), in stark contrast to both cisplatin and non-glycosylated analogues. This points out the role of the carbohydrate moiety not merely as a solubilizing group, but as a key contributor to tumor selectivity, likely through enhanced uptake mediated by cancer-associated glucose transporters or glycan-recognition pathways.

Expanding on this concept of metal-centered modulation within the same sugar-functionalized scaffolds, Al-Wasidi and collaborators [103] explored a markedly different approach by focusing on glucose-6-phosphate as the *O*-coordinating ligand and evaluating its complexation with a range of metal and non-metal ions: V(III) (**V1**), Ru(III) (**Ru19**), Au(III) (**Au18**), and Se(IV) (**Se1**). The authors successfully generated the metal-glycophosphate complexes, confirming bidentate coordination *via* the phosphate group and distinct geometries across the series, that is, octahedral for **V1** and **Ru19**, and square-planar for **Au18** and **Se1**. The most striking biological results came from the Au(III) complex, which exhibited remarkable cytotoxicity against HepG2 and MCF-7 cancer cell lines, with IC_50_ values substantially lower than the free ligand and comparable to cisplatin in HepG2. In addition to its anticancer potential, **Au18** and, to a lesser extent, the Ru(III) and Se(IV) analogues, also demonstrated noteworthy antibacterial activity, particularly against Gram-negative strains such as *Escherichia coli*, where their performance even surpassed standard antibiotics like ampicillin/sulbactam combination treatment. These effects were attributed to improved membrane permeability and altered lipophilicity induced by metal chelation, consistent with classical chelation theory [107]. This work highlights the flexibility of sugar-based ligands to serve as platforms for the design of bioactive metal complexes, and suggests that such systems, particularly those involving Au(III), hold potential for therapeutic development as multi-functional agents targeting both cancer and bacterial infections.

A further exploration of how sugar conjugation can fine-tune the selectivity and potency of metal-based anticancer agents was offered by Morales *et al.* [104], who reported on a family of Pd(II) (**Pd7**) and Cu(II) (**Cu1**) complexes derived from fluorinated *N*,*O* Schiff base ligands, as glycoconjugates bearing protected d-glucose units. *In vitro* testing revealed a clear superiority of the glycoconjugated species over their non-glycosylated counterparts. Notably, **Pd7**, here reported out of the family of palladium complexes, stood out as the most active and selective compound, exhibiting potent cytotoxicity across a panel of cancer cell lines, including prostate (PC-3), breast (MCF-7), colon (HCT-15), lung (SK-LU-1), leukemia (K-562), and glioblastoma (U-251), while maintaining significantly reduced toxicity towards non-cancerous COS-7 cells. Molecular docking studies provided further mechanistic insights, giving a possible explanation of their improved selectivity: Pd(II)-glycoconjugates show strong hydrogen-bond interactions with DNA, a feature absent in the weaker, more hydrophobic binding modes of the non-glycosylated analogs. This dual contribution, *i.e.,* improved cellular uptake *via* GLUT pathways and stronger engagement with biological targets, likely accounts for the superior therapeutic profile observed for these conjugates. The position of the trifluoromethyl group on the ligand also proved critical, with *ortho*-substituted derivatives such as **Pd7** outperforming *meta* and *para* analogues in both potency and selectivity.

The search for alternative platinum-group metal centers in the development of sugar-functionalized anticancer agents has recently been advanced by Bokor and co-workers [105], who introduced a novel class of half-sandwich organometallic complexes incorporating *C*-glucosaminyl azine ligands. In this study, metals such as Ru(II), Os(II), Ir(III), and Rh(III) were coordinated to arene or cyclopentadienyl moieties, forming well-defined organometallic scaffolds where the sugar-based azine ligand plays a key role in biological performance. The resulting complexes were evaluated for both antineoplastic and antimicrobial activities, positioning them at the intersection of oncology and infectious disease treatment. These ligands, derived from glucosamine and modified with various *N*-heterocycles (for simplicity, only pyridine ligands are here reported), were further decorated with benzyl (**Ru20**, **Os1**, **Ir1**, **Rh1**) or benzoyl protecting groups (**Ru21**, **Os2**, **Ir2**, **Rh2**), which significantly affected biological outcomes. Among the most potent compounds, **Ru20** and **Ir1** showed IC_50_ values in the low micromolar range against A2780 ovarian cancer cells. The therapeutic effect extended to other cell lines as well, including glioblastoma and pancreatic cancer, although the therapeutic window, the difference in cytotoxicity between cancerous and healthy cells, was moderate, suggesting the need for further refinement. The order of effectiveness for anticancer activity among the studied metals was: ruthenium > iridium > osmium > rhodium. What distinguishes this study is its dual biological scope. Several of the same metal-sugar complexes that were cytotoxic to cancer cells also exhibited strong bacteriostatic activity against multidrug-resistant Gram-positive strains such as methicillin-resistant *Staphylococcus aureus* and vancomycin-resistant *Enterococcus* [108]. In this context, ruthenium and osmium complexes, especially **Ru21** and **Os2**, were the most effective, with MIC values in the low micromolar range. Rhodium analogues, on the other hand, showed little activity in either domain. The study also reinforced the role of lipophilicity as a predictor of both cytotoxicity and antimicrobial potency, highlighting a key parameter for future optimization. While the precise molecular mechanism is yet to be elucidated, the strong performance of **Ru20–21** in particular suggests that sugar-appended azine ligands can serve as a versatile platform for designing dual-acting therapeutic agents.

An innovative angle on sugar-functionalized metal complexes is presented by Karban and co-workers [106], who explored how the combination of fluorinated glucosamine or galactosamine scaffolds with organometallic motifs, specifically ferrocene (**Fe1**) and ruthenium-tetrazene units (**Ru22–23**), can dramatically improve anticancer properties. To increase the cytotoxic potential of the sugar scaffold, the authors selectively fluorinated specific hydroxyl groups on glucosamine and galactosamine scaffolds, and these modified sugars were then further functionalized by attaching ferrocene or ruthenium-tetrazene fragments, either at the C^1^ or C^2^ positions of the sugar ring, using well-established synthetic procedures such as azide–alkyne cycloaddition. Among the resulting compounds, the ruthenium-tetrazene derivative **Ru22** and the ferrocene-bearing complex **Fe1** stood out for their low micromolar IC_50_ values (comparable to cisplatin) across a panel of cancer cell lines (A2780, SK-OV-3, MDA-MB-231). Both compounds also exhibited moderate selectivity over normal HEK-293 cells. In parallel, the α-anomer of the same ruthenium derivative (**Ru23**) showed slightly higher IC_50_ values but a markedly superior selectivity index, especially against the triple-negative breast cancer line MDA-MB-231, where it achieved a selectivity index exceeding 20. This suggests that despite its lower potency compared to the β anomer **Ru22**, the α-anomer **Ru23** may offer advantages in minimizing off-target toxicity, highlighting the importance of anomeric configuration not only for biological activity but also for therapeutic balance between efficacy and safety. Mechanistic studies revealed that **Ru22** induces apoptosis *via* DNA damage, with clear evidence of PARP cleavage, H2AX phosphorylation, and G2/M phase cell cycle arrest, accompanied by a substantial increase in ROS levels. The ferrocene complex **Fe1** also triggered apoptosis but without strong ROS generation, suggesting a distinct mechanism. Meanwhile, half-sandwich-type ruthenium complexes and galactosamine analogues exhibited minimal activity, likely due to poor cell permeability or suboptimal physicochemical features.

**Figure 14:**
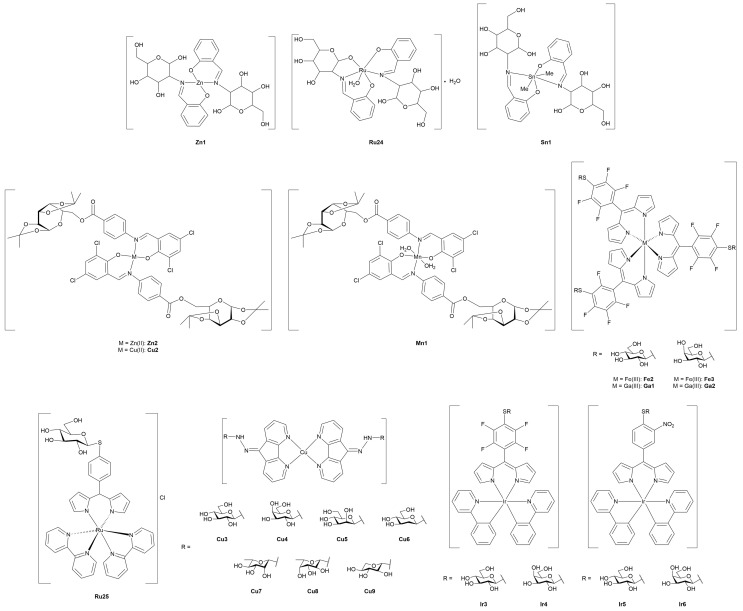
Chemical structures of some representative metal-glycoconjugates developed by Rashidi [109], Saha [113], Wiehe [114], Arias-Perez [115], Senge [116] and co-workers.

Other non-platinum-containing monometallic-glycoconjugates have been recently reported (Figure 14). For instance, Rashidi and collaborators contributed with the development of metal-glycoconjugates designed for selective cancer targeting, reporting on the synthesis and evaluation of Zn(II) (**Zn1**), Sn(IV) (**Sn1**), and Ru(III) (**Ru24**) complexes based on a Schiff base ligand derived from salicylaldehyde and d-glucosamine [109]. Their goal was to obtain targeted chemotherapeutic agents against colon cancer, leveraging the overexpression of GLUTs in malignant cells to improve drug selectivity and reduce systemic toxicity. The complexes’ biological activity was assessed on two colon cancer cell lines, HCT-116 and Caco-2, where the Ru(III) complex exhibited the most promising results. Although the ligand alone displayed some cytotoxicity, particularly on HCT-116 cells (IC_50_ = 21.73 μg/mL ≅ 76.7 μM), the ruthenium complex **Ru24** was more effective on Caco-2 (IC_50_ = 49.53 μg/mL ≅ 70.7 μM). The Zn(II) and Sn(IV) complexes proved significantly less potent, suggesting a strong dependence of activity on the nature of the metal center. A particularly compelling aspect of this study is the demonstration of GLUT-mediated cell uptake. Co-treatment with quercetin substantially reduced cytotoxicity, confirming that cellular entry of the glycoconjugates is, at least partially, dependent on GLUTs activity. This supports the hypothesis that glucosamine-based ligands can function not only as chelators but also as targeting vectors, directing metal complexes selectively into tumor cells. Mechanistically, the anticancer activity of the most active compounds was attributed to a dual mode of action: strong DNA binding (as shown by gel electrophoresis assays) and the induction of oxidative and nitrosative stress, evidenced by elevated markers such as nitric oxide, malondialdehyde (a marker of lipid peroxidation [110]), and lactate dehydrogenase (a marker of cell membrane damage [111]). Docking studies further reinforced the biological relevance of the Ru(III) complex, which showed favorable interactions with cyclin-dependent kinase 2 (CDK2), a key regulator of cell cycle progression often upregulated in colorectal cancers [112]. Interestingly, the study also uncovered antibacterial activity, particularly for the **Zn1** complex, against *Helicobacter pylori*, where the Zn(II) compound’s effect was comparable to that of amoxicillin. This multi-target profile, anticancer and antibacterial, echoes a trend observed in other metal-glycoconjugates discussed in this section.

The integration of sugar moieties into metal complexes to improve both biocompatibility and anticancer performance has been further explored by Saha *et al.* [113], who reported on a new monosaccharide-linked Schiff base ligand and its corresponding Zn(II) (**Zn2**), Cu(II) (**Cu2**), and Mn(II) (**Mn1**) complexes. In this case, the carbohydrate, derived from a protected galactose unit, is covalently bound to a Schiff base framework formed from 3,5-dichlorosalicylaldehyde and 2-aminobenzoic acid. The biological evaluation revealed substantial antiproliferative activity across three cancer cell lines, namely A549, HeLa and HT29. All complexes exhibited IC_50_ values in the low micromolar range (8.8–23 µM), comparable to cisplatin. **Cu2** and **Mn1** complexes showed slightly superior performance on lung and colorectal cancer cells, suggesting metal-specific contributions to cytotoxicity. Mechanistic studies pointed clearly toward apoptosis as the primary mode of cell death, supported by DNA fragmentation patterns, nuclear morphology changes (Hoechst staining), and acridine orange/ethidium bromide assays. Crucially, all three metal complexes significantly increased intracellular ROS levels, providing a mechanistic link between metal-induced oxidative stress and apoptotic signaling. The absence of necrosis markers further supports a controlled, non-inflammatory cytotoxic response, desirable for minimizing off-target effects [117].

A complementary approach to metal-glycoconjugation is presented by Wiehe and collaborators through the development of a series of tris(dipyrrinato) metal complexes functionalized with carbohydrate moieties via post-synthetic modification [114]. Starting from Ga(III)-, Fe(III)-, and Ru(II)-dipyrrinato precursors bearing a pentafluorophenyl substituent, the authors introduced thioglucose (**Ga1**, **Fe2**, **Ru25**) and thiogalactose (**Ga2**, **Fe3**) residues through nucleophilic aromatic substitution, providing a modular route for sugar attachment that preserves the integrity of both the carbohydrate and the metal-ligand framework. The post-functionalization approach proved more efficient than pre-functionalization, which would otherwise compromise thiol reactivity during oxidative steps in ligand assembly. Biological evaluations showed a marked phototoxicity of the Ga(III) derivatives, which induced significant light-dependent cytotoxicity in multiple cancer cell lines (A-253, A-431, CAL27, HT-29), as well as antibacterial activity against *Staphylococcus aureus*. In contrast, the Fe(III) analogues were inactive under both light and dark conditions, and non-glycosylated controls failed to reproduce the observed effects, underscoring the critical role of both metal identity and carbohydrate presence. While the precise mechanism of action remains to be clarified, a photoinduced generation of ROS is likely involved, and the sugar units may enhance uptake and/or affect subcellular localization. The successful extension of this glycosylation method to ruthenium complex **Ru25**, although without full biological characterization, demonstrates the general applicability of the synthetic route and the adaptability of the proposed platform.

In a separate work [115], Arias-Perez *et al.* developed a family of Cu(II) complexes based on 4,5-diazafluorene *N*-glycopyranosyl hydrazone ligands, exploring their potential as cytotoxic agents and DNA-targeting compounds. These ligands were obtained through a direct condensation reaction, yielding stable and stereoselective cyclic hydrazone derivatives without the need for protecting groups. Subsequent complexation with Cu(II) led to a series of compounds (**Cu3–9**) incorporating various sugar units, including glucose, deoxyglucose, rhamnose, fucose, and xylose. Biological studies showed that the cytotoxic activity was strongly influenced by the carbohydrate moiety: while the glucose-based complex was inactive, the fucose (**Cu8**), rhamnose (**Cu7**), and deoxyglucose (**Cu6**) derivatives recorded low micromolar IC_50_ values against HeLa and PC3 cancer cell lines, in some cases outperforming cisplatin. These effects were not observed for the free ligands, confirming that metal coordination is essential for bioactivity. The DNA-binding experiments using fluorescence-based Förster Resonance Energy Transfer (FRET) melting assays showed that the Cu(II) complexes with *N*-glycopyranosyl hydrazone ligands were able to stabilize G-quadruplex DNA structures, which are known to play important roles in cancer-related genomic regulation. The authors suggested that this selectivity for G-quadruplexes may indeed contribute to the observed anticancer activity of the most cytotoxic compounds.

A further contribution to the field of glycosylated iridium complexes was brought by Senge and collaborators [116], who developed a complete library of Ir(III)-dipyrrinato complexes as photoactivatable agents for both PDT and antimicrobial photodynamic inactivation. Upon white-light irradiation, complexes **Ir3–6** displayed potent and selective phototoxicity against cancer cell lines A-431 and HT-29, with minimal dark toxicity. The most active was the galactosylated complex **Ir4**, which not only retained high photodynamic efficacy but also demonstrated improved water solubility and cellular uptake, likely due to the hydrophilic and targeting properties of the carbohydrate moiety. Interestingly, many of these compounds also showed significant activity against bacteria, especially *Staphylococcus aureus*, with complete inactivation at low micromolar concentrations upon irradiation. The galactose-functionalized complex **Ir4** was particularly effective even in complex media containing serum, an important step toward clinical applicability.

#### 3.4.2. Bimetallic-Glycoconjugates

**Figure 15:**
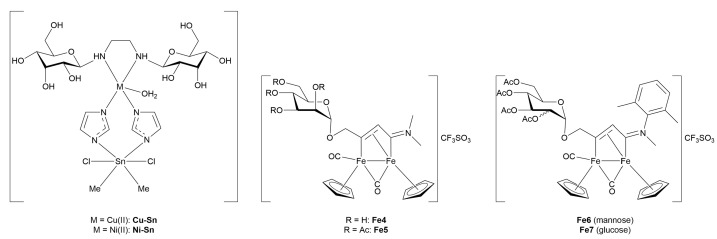
Chemical structures of some representative multi(hetero)-metal-glycoconjugates developed by Tabassum [119], Marchetti [120] and co-workers.

An additional frontier in the field of metal-based glycoconjugates is represented by multi-metallic complexes, which incorporate within the same molecular scaffold two or more metal centers, either of the same type or different. In a therapeutic context, multi(hetero)metallic systems offer a promising strategy to improve biological efficacy, improve selectivity, or introduce synergistic mechanisms of action that are often inaccessible with conventional monometallic compounds (Figure 15) [118].

This concept is effectively demonstrated in the study carried out by Tabassum and co-workers [119], which reports on the synthesis and biological evaluation of two novel carbohydrate-based heteronuclear complexes combining Sn(IV) with either Cu(II) (**Cu-Sn**) or Ni(II) (**Ni-Sn**). Both complexes are built upon an *N*-glycoside ligand, exploiting the high metabolic uptake of sugars by tumor cells to increase selectivity. The compounds were fully characterized by spectroscopic and computational methods, and their interaction with DNA was assessed through both *in vitro* binding studies and molecular docking. Among the two, the **Cu-Sn** complex demonstrated particularly promising biological activity, showing high affinity for DNA and the ability to induce hydrolytic cleavage. Moreover, it inhibited the activity of topoisomerase Iα at low micromolar concentrations, blocking the relaxation of supercoiled DNA, a mechanism that is crucial for cancer cell proliferation [121]. Moreover, it showed strong antiproliferative activity across a panel of human cancer cell lines, with GI_50_ values below 10 µg mL^−1^, indicating substantial potency. Confocal microscopy further confirmed that the compound localizes within the nucleus of HeLa cells and binds DNA in the minor groove. Molecular modeling supported the experimental findings: docking simulations suggested that it binds to the active site of topoisomerase I and interacts with the ribofuranose moiety of a guanine residue, likely obstructing the religation step of the enzyme and thereby halting DNA replication. In contrast, the **Ni-Sn** complex was synthesized and characterized but not extensively evaluated biologically.

Another example of glycoconjugates incorporating multiple metal centers has been recently provided by Marchetti and co-workers [120], who developed a series of diiron vinyliminium complexes bearing different carbohydrate units. In this study, the authors explored the combination of a bimetallic iron scaffold, known for its redox activity and potential to induce oxidative stress in cancer cells, with carbohydrate moieties such as mannose, glucose, and fructose, aiming at enhancing aqueous solubility and selective uptake *via* GLUTs. The glycoconjugates here reported, namely **Fe4–7**, were synthesized through regioselective insertion of sugar-derived propargyl glycosides into the vinyliminium core, and compared with the non-glycosylated reference compounds. The resulting complexes exhibited varying degrees of water solubility and good stability in physiological-like conditions, especially when sugars were appropriately protected. Biological evaluation on several cancer (CT26, U87, MCF-7) and non-cancerous (RPE-1) cell lines showed that the more hydrophilic glycoconjugates (**Fe4** and **Fe5**) displayed limited cytotoxicity, likely due to poor membrane permeability. In contrast, the more hydrophobic analogues (**Fe6** and **Fe7**) demonstrated moderate cytotoxicity, though without improved selectivity over healthy cells. Importantly, glucose deprivation did not enhance cellular uptake or cytotoxicity of the glycosylated complexes, suggesting that GLUT-mediated transport was not involved in this case. Furthermore, migration assays indicated no significant anti-metastatic effects at sub-cytotoxic concentrations. The mechanism of action is believed to involve intracellular degradation of the diiron core with subsequent iron release, leading to disruption of redox homeostasis rather than sugar-mediated targeting. Although the incorporation of sugars did not result in improved biological activity, this study highlights the synthetic versatility of the diiron vinyliminium platform and underscores the importance of balancing hydrophilicity and membrane permeability when designing carbohydrate-functionalized metal-based therapeutics.

In summary, the studies discussed in this section illustrate the wide chemical diversity and design strategies available when exploring metals beyond those traditionally employed in glycoconjugated systems. Whether through metal substitution within a common scaffold, bimetallic architectures, or incorporation of less conventional metals, these works highlight both the opportunities and challenges in modulating biological activity through metal choice, ligand environment, and glycosylation.

## 4. Metal-Glycoconjugates for Tumor Imaging Applications

**Figure 16:**
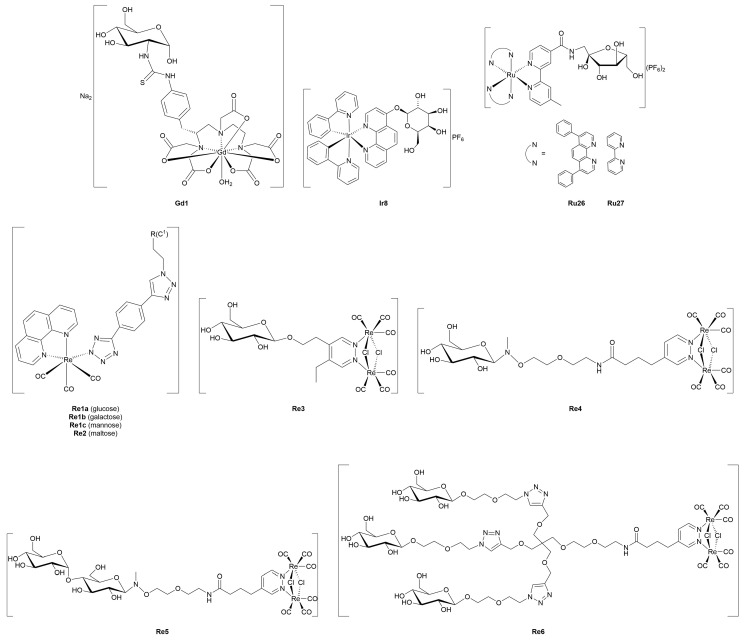
Chemical structures of some representative metal-glycoconjugates for bioimaging applications developed by Ardestani [125], Lo [126], Ma [127], Plush [128], Panigati [129] and co-workers.

Magnetic resonance imaging (MRI) is one powerful non-invasive technique available for cancer diagnosis, offering excellent spatial resolution and soft tissue contrast [122]. Nonetheless, its diagnostic potential heavily depends on the use of contrast agents, particularly those based on the Gd(III) center, which improves image quality by altering local relaxation times of water protons [123]. Despite their widespread clinical application, conventional Gd(III) chelates, such as the aforementioned Magnevist, suffer from limitations including (too) rapid clearance, insufficient cellular uptake, and limited selectivity for malignant tissues [124]. In order to overcome those drawbacks, recent research has been focusing on molecularly targeted MRI probes, in particular those conjugated to biomolecules—including carbohydrates—to improve selective uptake into cancer cells *via* GLUT-mediated pathways (Figure 16).

The study by Ardestani and co-workers [125] exemplifies this approach by presenting a glucose-functionalized gadolinium agent with promising properties for early cancer detection. The researchers reported on the synthesis and *in vitro* evaluation of a novel Gd(III)-based contrast agent functionalized with a glucose derivative (**Gd1**). The key innovation lies in the conjugation of d-glucosamine to a widely used chelator, para-isothiocyanatobenzyl-diethylenetriaminepentaacetic acid, to improve selective uptake into cancer cells *via* GLUT-mediated pathways. The compound was tested on breast cancer (MCF-7) and normal kidney (HEK 293) cells to assess antiproliferative activity, selectivity and uptake. Cytotoxicity assays confirmed that the compound is non-toxic to normal cells, while a moderate reduction in cancer cell viability was observed at higher concentrations, likely a reflection of greater internalization. Cellular uptake studies showed that the glucose-functionalized agent was taken up by 67% of MCF-7 cells, compared to only 5% for Magnevist, accounting for a 14-fold improvement. From a diagnostic perspective, the new agent demonstrated high relaxivity values, comparable with the reference drug Magnevist. These properties translate into strong and clear MRI signals, a key feature for clinical tumor imaging. Mechanistically, the selective uptake is attributed to GLUT-mediated internalization, conceptually analogous to [^18^F]-2-fluoro-2-deoxy-d-glucose used in PET imaging [38], but implemented here in an MRI context. Although further *in vivo* validation is necessary, this study paves the way for the development of glucose-targeted molecular imaging tools for tumor diagnosis.

An alternative approach to optical imaging diagnostic methods relies on the development of phosphorescent probes for (confocal) fluorescence microscopy. Intriguingly, luminescent transition metal complexes, especially those containing Ru(II), Ir(III) and Rh(I) ions, are amongst the most promising platforms for live-cell imaging due to their favorable photophysical characteristics, that is, long-lived emission, resistance to photobleaching, and tunable excitation/emission profiles in the visible range [130]. The ability to chemically modify these complexes with biologically active moieties, such as sugars, allows fine control over their cellular uptake, intracellular localization, and selectivity for specific cell types. The following recent examples are specifically concerned with the development of metal-based glycoconjugates designed for confocal fluorescence imaging, in which the attachment of carbohydrates allows the strategic targeting of cancer-related metabolic pathways.

Lo and collaborators investigated a series of luminescent Ru(II)-polypyridine complexes functionalized with d-fructose as potential selective imaging agents in cancer cells [126]. The rationale of this research stems from the growing interest in the design of non-toxic fluorescent probes that can exploit cancer-specific metabolic features, particularly the elevated expression of fructose transporters (*e.g.,* GLUT5) in certain tumor types like breast cancer. Four complexes were synthesized by combining Ru(II) precursors with bipyridine ligands, either bearing a d-fructose pendant (**Ru26–27**) or an ethyl substituent as a control. Structural and photophysical characterizations confirmed that all compounds exhibit strong and stable luminescence in the orange-red region (λ_em_ = 628–643 nm), with only modest spectral shifts between fructose-modified and unmodified analogues. The introduction of the sugar unit substantially decreased the overall lipophilicity, impacting the extent of cellular uptake. All compounds demonstrated low cytotoxicity in MCF-7 breast cancer cells, maintaining over 70% cell viability even at high concentrations, making them suitable for non-disruptive bioimaging applications. Confocal microscopy revealed distinct localization patterns across different cell lines. In MCF-7 cells, both **Ru26** and its corresponding aglycone stained the plasma membrane, as confirmed by co-localization with a membrane-specific dye. In HeLa cells, instead, while **Ru26** retained membrane accumulation, its non-glycosylated counterpart migrated to the mitochondria. Finally, in MDCK and 3T3 cells, both complexes showed more diffuse cytoplasmic distribution, suggesting that cell-type-specific uptake and retention mechanisms are at play. A major contribution of the object research work was the demonstration that uptake of the d-fructose-containing **Ru26** complex occurs through a GLUT5-mediated mechanism: competition assays performed in the presence of excess d-fructose significantly reduced the uptake of the Ru(II)-glycoconjugate, indicating transporter saturation, further confirmed with flow cytometry. This selective mechanism supports the potential use of complex **Ru26** as a targeted imaging probe for cells that overexpress GLUT5, such as MCF-7 breast cancer cells.

Another key contribution to the field was provided by the study of Ma *et al.* [127], which reports on the development of a novel Ir(III)-based luminescent probe (**Ir8**) specifically designed to visualize β-galactosidase activity in ovarian carcinoma cells. β-Galactosidase is an enzyme well known for its role in gene regulation, especially within the lactose operon system, and as a hallmark of cellular senescence [131], and it has recently gained recognition as a clinically significant biomarker for primary ovarian cancer [132]. However, conventional β-galactosidase detection techniques, such as immunostaining or colorimetric assays, often suffer from limited sensitivity, complex sample preparation, and poor compatibility with live-cell imaging [133]. Fluorescent probes have improved in this regard but are still hindered by biological autofluorescence and, crucially, by a lack of specificity for cancer cells over normal tissues [134]. To address these limitations, the authors designed the aforementioned Ir(III) complex **Ir8**, featuring a galactose moiety that would act as both a molecular trigger and a luminescence quencher. In fact, in its intact state, the probe remains weakly emissive. Upon enzymatic cleavage of the galactose unit by β-galactosidase, the complex is converted into its active form, which exhibits strong yellow phosphorescence. This “light-up” mechanism is further improved by the use of time-resolved emission spectroscopy (TRES), which allows selective detection of the long-lived emission of the iridium probe while eliminating short-lived background fluorescence, an intrinsic challenge in biological imaging [135]. Biological evaluation demonstrated that the probe responds selectively to β-galactosidase *in vitro* and in cellular models. Notably, the probe maintained its performance even in complex biological samples, such as human serum, and in the presence of autofluorescent dyes, highlighting the advantage of its long-lived emission. In cellular studies, ovarian carcinoma cell lines (SKOV3 and OVCAR3) showed strong luminescence after treatment with the probe, while normal cells (HEK-293T and HUVEC) exhibited negligible signals. This selective activation was further validated by pre-incubating SKOV3 cells with d-galactose, a competitive inhibitor of β-galactosidase, which markedly reduced the luminescent response. These findings confirm that the probe operates *via* a β-galactosidase-specific mechanism and effectively discriminates between malignant and non-malignant cells. Equally important is the probe’s low cytotoxicity under imaging conditions, which ensures compatibility with live-cell applications. Together, these features position **Ir8** as a powerful molecular tool for non-invasive, real-time imaging of enzymatic activity in cancer cells.

Plush and co-workers have recently explored a distinct and forward-looking strategy for live-cell imaging using metal-based glycoconjugates by presenting a comprehensive study on a platform of neutral Re(I) complexes, specifically developed to overcome some major limitations of traditional cationic metal probes, such as mitochondrial accumulation and, in some cases, elevated cytotoxicity [128]. The authors generated four glycoconjugated Re(I)-carbonyl complexes—namely, **Re1a** (glucose), **Re1b** (galactose), **Re1c** (mannose), and **Re2** (maltose)—all exhibiting strong metal-to-ligand charge transfer (MLCT) emission centered near 584 nm and, thus, excitable with standard 405 nm lasers commonly used in microscopy. Their long emission lifetimes and moderate quantum yields make them well-suited for time-resolved imaging, allowing endogenous short-lived autofluorescence to be selectively excluded from detection windows. These photophysical properties, combined with their chemical stability, make them a valid imaging tool for biological studies. Their biological evaluation was carried out in H9c2 cardiomyoblasts, a cell line chosen for its heavy dependence on carbohydrate metabolism. Uptake studies revealed that the glucose-based complex **Re1a** showed the highest internalization, followed by the mannose (**Re1c**), maltose (**Re2**), and galactose (**Re1b**) counterparts. Interestingly, this uptake did not correlate exclusively with lipophilicity, suggesting that different sugar moieties also play a role, perhaps by interacting with cellular membranes. Notably, competition experiments using excess d-glucose showed no inhibition of cell uptake, indicating that GLUT transporters may not be involved. Subcellular localization, observed through confocal microscopy, was markedly different from that of traditional positively charged complexes. The neutral Re(I)-glycoconjugates accumulated predominantly in the endosomal and lysosomal compartments, with additional signal in the endoplasmic reticulum, especially for the mannose and maltose derivatives. As expected after removal of the cationic charge, no mitochondrial localization was observed. Cytotoxicity assays confirmed that all complexes are relatively non-toxic, showing only a moderate effect on cell viability after 24 h at higher concentrations. There were no signs of acute cell stress or membrane damage, indicating that those compounds have potential for live-cell imaging, inducing minimal perturbation to the system under study.

Finally, Panigati and collaborators [129] presented a significant advancement in the development of sugar-functionalized Re(I) complexes for live-cell imaging. The research work introduced a new family of dinuclear Re(I)-glycoconjugates (**Re3–6)** designed with the dual intent of maximizing biocompatibility and imaging performance. In contrast to earlier Re(I)-based probes, which often suffered from cytotoxicity and poor solubility, such newly developed complexes incorporate sugar moieties to increase water solubility, reduce toxicity, and direct intracellular localization. The authors focused on HeLa cells to evaluate the intracellular behavior of their glycosylated Re(I) complexes. Synthetically, the study showcased both traditional glycosylation strategies and neo-glycorandomization, a chemoselective method that allows rapid attachment of unprotected sugars to aminooxy-functionalized scaffolds without the need for protecting groups. This modularity allowed the preparation of several glyco-functionalized Re(I) complexes, including multivalent constructs bearing multiple glucose units. These multivalent systems were introduced to probe the influence of sugar valency on solubility, self-assembly, and biological activity. From the photophysical point of view, the complexes displayed intense yellow-orange emission between 570 and 594 nm, with good quantum yields and stability under irradiation. Although glycosylation did not significantly alter the intrinsic electronic structure of the metal cores, it did impart amphiphilic character, promoting the formation of supramolecular aggregates in aqueous environments. Interestingly, this aggregation led to increased photophysical properties in some cases (*e.g.,* for complexes **Re4** and **Re5**), such as prolonged excited-state lifetimes, but could also result in emission quenching, particularly in the most heavily glycosylated species (**Re6**), highlighting the delicate balance between multivalency and performance. Live-cell imaging experiments revealed rapid and efficient uptake of the complexes by HeLa cells, with intracellular accumulation observed within minutes. Once internalized, the complexes preferentially localized in the cytoplasm, with strong selectivity for the endoplasmic reticulum. This organelle-specific targeting was especially pronounced in compounds bearing more hydrophilic sugars, such as the maltose-bearing complex **Re5**, suggesting that both the nature of the sugar and the physicochemical properties of the complex affect its trafficking within cells. Notably, none of the complexes accumulated in the nucleus or cytoskeletal structures. All complexes exhibited very low toxicity, with cell viability exceeding 93% after 1-h incubation at concentrations up to 50 µM. Among the series, compounds **Re4** and **Re5** emerged as the most effective imaging agents, combining high luminescence intensity, rapid uptake, preferential accumulation in the endoplasmic reticulum, and minimal toxicity. Meanwhile, compound **Re6** demonstrated that increasing valency may improve solubility but may also lead to aggregation-induced quenching, limiting its usefulness for fluorescence-based applications.

## 5. Therapeutic Metal-Glycoconjugates Beyond Oncological Applications

### 5.1. Antimicrobial, Antiviral, and Antiparasitic Metal-Glycoconjugates

**Figure 17:**
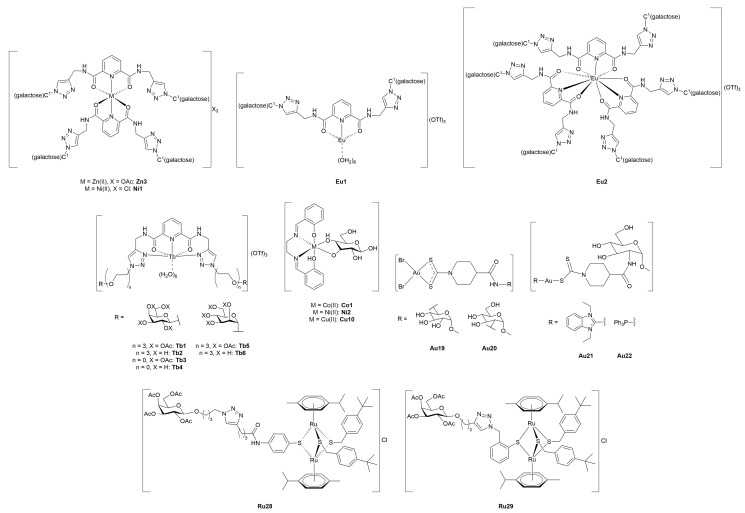
Chemical structures of some representative metal-glycoconjugates for antimicrobial, antiviral and antiparasitic applications developed by Byrne [141,142], Hesien [143], Ott & Ronconi [144,145], Păunescu [146] and co-workers.

In recent years, the therapeutic potential of metal-glycoconjugates has expanded beyond oncology, showing promise in the treatment of infectious diseases [136]. The growing threat of antimicrobial resistance and the global impact of viral outbreaks like COVID-19 have highlighted the urgent need for novel therapeutic strategies [137,138]. In this context, glycoconjugate metal complexes have emerged as versatile candidates capable of targeting key molecular mechanisms in pathogens, including adhesion, enzymatic activity, and redox balance [139,140]. This section explores recent developments in the use of metal-glycoconjugates as antibacterial, antifungal, antiviral, and antiparasitic agents. The selected studies illustrate a wide range of mechanisms: from blocking microbial adhesion and biofilm formation to the luminescent sensing of bacterial lectins and the inhibition of viral proteases and protozoan proliferation, making this class of compounds a versatile platform for developing innovative anti-infective therapies (Figure 17).

Byrne and collaborators [141] have just reported on an innovative chemical strategy to combat infections caused by *Pseudomonas aeruginosa* and *Candida albicans*, two high-priority pathogens according to the World Health Organization due to their increasing resistance to conventional antimicrobials [147]. Instead of aiming at killing these pathogens directly, a strategy that often drives the development of resistance [148], this study targets adhesion to host tissues, a key virulence factor [149]. This process, mediated by lectins in bacteria and adhesins in fungi, is essential for colonization and biofilm formation [150,151]. By blocking these interactions, pathogens can be disarmed rather than destroyed, potentially avoiding selective pressure that leads to antimicrobial resistance [152].

The researchers focused on a digalactoside glycoconjugate ligand, designed to bind specifically to the galactophilic bacterial lectin LecA [153]. To enhance its biological effect, the ligand was coordinated to various metal ions, Eu(III) (**Eu1–2**), Ni(II) (**Ni1**), and Zn(II) (**Zn3**), according to the idea that metal coordination could enable multivalent sugar display and improved molecular topology, thus increasing antiadhesive efficacy. Binding studies showed that the ligand itself binds LecA with moderate affinity (*K*_d_ ~9.6 μM), sufficient to serve as a functional scaffold. Metal complexation was found to dramatically impact biological activity. While **Ni1** and **Zn3** complexes had limited or modest effects, the Eu(III) derivatives, particularly **Eu1**, significantly inhibited *Pseudomonas aeruginosa* biofilm formation by up to 60% at 0.1 mM without displaying bactericidal activity. These findings underline that bioactivity is not solely dictated by lectin affinity, but also by the precise geometry and coordination environment imposed by the metal center. A similar antiadhesive effect was observed for *Candida albicans*. The trivalent Eu(III) complex **Eu2** inhibited yeast adhesion to human buccal epithelial cells by up to 63%, matching or exceeding previous best-in-class antiadhesive agents. The free ligand achieved 35–47% inhibition, while **Zn3** and **Ni1** complexes showed moderate to low efficacy, the latter also displaying cytotoxicity to the yeast. Notably, the Eu(III) complexes caused visible clumping of yeast cells, consistent with a multivalent clustering mechanism that may sterically block adhesion. Mechanistic insights from DFT modeling suggested that metal complexation subtly affects the spatial orientation and flexibility of the galactose moieties, changes that likely explain the observed variations in biological activity. In conclusion, this work demonstrates that coordination of a suitably designed glycoconjugate ligand to a specific metal center, in this case Eu(III), can dramatically increase its antiadhesive and anti-biofilm activity. Importantly, these effects are achieved without killing the pathogens, supporting the development of non-lethal, resistance-sparing therapeutics. These metal-glycoconjugate complexes represent a promising platform for novel anti-infective strategies targeting virulence rather than viability, and could serve both as therapeutics and, potentially, as diagnostic tools if functionalized further.

Building on their previous work, Byrne *et al.* further explored the exploitation of glycoconjugate chemistry and metal coordination for the development of a novel diagnostic platform based on Tb(III) luminescent probes [142]. In this follow-up study, they addressed a critical unmet need in infectious disease diagnostics, that is, the rapid, selective, and label-free detection of pathogenic bacteria such as *Pseudomonas aeruginosa*. The target of this sensing approach is again LecA, as in the previous work. While traditional diagnostic strategies often rely on tagging such proteins with fluorescent labels or culturing bacteria over long periods, this work introduces an elegant alternative: a luminescent glycoconjugate complex that emits a signal only upon binding to its unlabeled protein target in solution. The system is built around lanthanide coordination, specifically Tb(III), which is known for its sharp emission bands, long lifetimes, and reduced background interference in biological settings. The authors designed multivalent 2,6-dipicolinic acid-based ligands decorated with either galactosides (**Tb1–4**) or mannosides (**Tb5–6)** through flexible linkers, in order to enable strong, multivalent interactions with lectins. These probes retained their lectin-binding capacity: the galactoside-containing complex **Tb2** showed selective, micromolar binding to LecA, and its luminescence at 545 nm increased threefold upon exposure to the protein. This “switch-on” effect was both selective and dependent on proper protein folding, as no luminescence enhancement was observed in the presence of denatured LecA or unrelated proteins. The selectivity of the system extended to sugar-lectin pairing: while **Tb2** responded specifically to LecA, the mannoside analogue **Tb6** was activated only by ConA, a mannose-binding lectin [154]. Furthermore, the luminescent signal was not quenched by excess free sugar, suggesting that the multivalent architecture of the ligand contributes to its strong, specific binding. Importantly, the complexes were biologically inert: they showed no antimicrobial activity, did not interfere with *Pseudomonas aeruginosa* growth or biofilm formation, and are thus well-suited for diagnostic use without adding selective pressure for resistance.

Continuing on the exploration of bioactive metal-glycoconjugates, the study by Hesien *et al.* [143] introduces a complementary approach focused on oxidative stress and antimicrobial defense, rather than adhesion or sensing. This work focuses on the synthesis and characterization of mixed-ligand metal(II) complexes combining a classic Schiff base ligand (*N*,*N*′-bis(salicylidene)ethylenediamine, salen) with d-glucose, coordinated to Co(II) (**Co1**), Ni(II) (**Ni2**), and Cu(II) (**Cu10**) ions. The resulting compounds have been evaluated not only for antimicrobial effects but also for their antioxidant properties and ability to mimic key metalloenzymes, namely superoxide dismutase (SOD) and catalase. The rationale behind this molecular design relies on the well-documented coordination ability and redox versatility of Schiff bases like salen, combined in this case with the glucose moiety. The integration of these two ligand types creates complexes with potential dual functionality: antimicrobial activity through improved cell permeability and metal-driven enzyme inhibition, and antioxidant behavior *via* radical scavenging and redox catalysis. Biological assays revealed that all complexes exhibit strong antimicrobial activity against both Gram-positive and Gram-negative bacteria, as well as fungi like *Candida albicans* and *Aspergillus flavus*. The Cu(II) complex **Cu10** turned out to be the most potent, sometimes outperforming standard drugs such as tetracycline and ciprofloxacin. In terms of antioxidant performance, the Cu(II) complex again stood out, showing high SOD-like activity, achieving up to seven times the efficiency of commercial mimics like EUK-8 [155]. Catalase-mimicking activity was likewise significant, with all three metal complexes showing robust decomposition of hydrogen peroxide. These findings support further exploration of such metal-glycoconjugate systems in the development of therapeutics for infections and oxidative stress-related conditions, offering a distinct but complementary avenue to antiadhesive or diagnostic approaches.

Following the outbreak of the COVID-19 pandemic, Ott and co-workers carried out a comprehensive assessment of the potential application of several metal complexes in the antiviral field with a large-scale screening effort aimed at combating SARS-CoV-2 [144]. Their study focuses on identifying metal-containing compounds capable of disrupting two critical viral processes, namely, the interaction between the viral spike protein and the human ACE2 receptor (essential for viral entry) [156], and the activity of the viral papain-like protease PL^pro^ (crucial for replication and immune evasion) [157]. Among the over 100 compounds tested, including known metallodrugs and newly synthesized complexes, a series of gold-glycoconjugates, including two Au(III) (**Au19–20**) and two Au(I) (**Au11–22**) derivatives, previously developed by the Ronconi group [145], emerged as standout performers. In this context, glycosylation served not only to improve solubility and biocompatibility but also to fine-tune the interaction of the metal complex with viral enzymes and host components. All four tested compounds displayed sub-micromolar IC_50_ values against PL^pro^, with **Au20** emerging as the most potent (IC_50_ = 0.09 μM), followed by **Au19** (0.21 μM), **Au22** (0.14 μM), and **Au21** (0.41 μM). Notably, **Au19** and **Au20** were more selective for SARS-CoV-2 PL^pro^ than for its SARS-CoV counterpart, indicating virus-specific interaction likely influenced by both metal coordination and glycan structure. Beyond biochemical assays, selected compounds were tested in cell-based antiviral models, where **Au19** and **Au20** completely blocked SARS-CoV-2 replication at higher concentrations (500 μM). While lower dose activity was less pronounced, these results suggested a strong potential for further optimization in terms of stability, bioavailability, or formulation. In conclusion, **Au19** and **Au20** stand out as highly potent and selective inhibitors of SARS-CoV-2 PL^pro^ values in the nanomolar range. These findings point to gold-glycoconjugates as a promising platform for the next generation of metal-based large-spectrum antivirals, particularly those targeting coronavirus proteases.

Expanding beyond bacterial and viral targets, metal-glycoconjugate systems have also shown promise against parasitic infections. In this context, the work carried out by Păunescu and collaborators [146] explored a family of organometallic glycoconjugates with potent activity against protozoan parasites. The study builds on the known antiparasitic potential of dinuclear trithiolato-bridged Ru(II)-arene complexes, a class of compounds previously shown to be active against *Toxoplasma gondii* and *Trypanosoma brucei* [158,159]. The authors sought to investigate how conjugation with carbohydrates would influence the properties of these ruthenium complexes, with the aim of improving water solubility, biological compatibility, or even targeting through sugar–transporter interactions in parasites. A series of novel metal-glycoconjugates was synthesized, featuring mono- and disaccharide units (*e.g.,* glucose, galactose, lactose) attached *via* ester, amide, or triazole linkers to the diruthenium scaffold. These structural variations allowed the authors to assess how different linkage types and sugar identities affect biological activity. The antiparasitic activity of the new glycoconjugates was evaluated *in vitro* against *Toxoplasma gondii* tachyzoites. Several compounds demonstrated nanomolar EC_50_ values, with the most active conjugates outperforming both the parent ruthenium complexes and standard drugs like pyrimethamine. Cytotoxicity assays against human foreskin fibroblasts (HFF) were also conducted to assess selectivity. Many of the active compounds displayed a favorable therapeutic index, exhibiting strong antiparasitic effects at concentrations that were not harmful to host cells. This is particularly important given the narrow safety margins of many current antiparasitic agents. Overall, the most active glycoconjugates were the galactose-based compounds **Ru28** and **Ru29**, which returned both superior antiparasitic effects and low human cell toxicity. Mechanistically, while the precise mode of action remains to be elucidated, the authors hypothesize that sugar conjugation may influence cellular uptake, intracellular localization, or interaction with biomolecular targets in the parasite, while the trithiolato-bridged diruthenium core is believed to disrupt redox homeostasis or key metabolic pathways in the parasite. In conclusion, the authors demonstrated that glycosylated Ru(II)-arene complexes retain, and in some cases surpass, the antiparasitic potency of their non-glycosylated counterparts. The integration of carbohydrates improves solubility and may improve selectivity, paving the way for further development of glycoconjugated metal complexes as next-generation antiparasitic agents. This work adds yet another dimension to the versatility of metal-glycan conjugates in biomedical applications.

### 5.2. Metal-Glycoconjugates for Neurological Disorders

**Figure 18:**
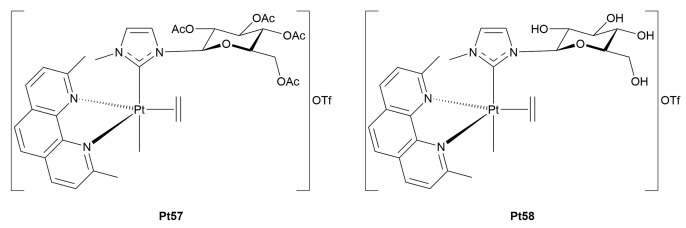
Chemical structures of some representative metal-glycoconjugates with potential applications in the treatment of neurological disorders developed by Marasco [165] and co-workers.

As previously pointed out, while most efforts in metal-glycoconjugate research have been centered around oncological applications, recent studies have highlighted the broader therapeutic potential of these compounds in less explored fields such as neurodegeneration and oxidative stress [160,161]. In particular, neurodegenerative diseases such as Alzheimer’s offer an opportunity for metal-based intervention due to the known involvement of metal ions in amyloid aggregation pathways [162,163]. This last section highlights one such application, where Pt(II)-glycoconjugates are explored as inhibitors of amyloid-β (Aβ) aggregation (Figure 18), a hallmark of Alzheimer’s pathology [164].

In this work [165], Marasco and co-workers examined two rationally designed Pt(II) complexes bearing glucose-derived ligands (**Pt57–58**) for their ability to disrupt the aggregation of Aβ peptides. The focus here was on *C*-terminal fragments of Aβ (Aβ21–40 and Aβ25–35), which are particularly prone to aggregation [166], yet less commonly targeted than the *N*-terminal region [167]. Two Pt(II) scaffolds were compared: a peracetylated version (**Pt57**), which is more hydrophobic, and a deprotected analogue (**Pt58**), which is more hydrophilic and biocompatible. Through a series of biophysical methods, including Thioflavin T fluorescence, spectroscopy, mass spectrometry, and microscopy, the authors demonstrate that **Pt58** strongly inhibits peptide aggregation, particularly for Aβ25–35, reducing fibril formation by up to 70%. For Aβ21–40, **Pt58** inhibitory effect was clearly dose-dependent: aggregation was decreased by 60% at a 1:1 ratio and up to 90% at a 1:5 peptide-to-metal complex ratio. ESI-MS experiments confirmed the formation of stable adducts between **Pt58** and both peptides, showing specific stoichiometries (including 1:1 for both peptides, and a unique **Pt58**:2Aβ25–35 adduct for the shorter fragment). The data indicate direct interaction, with the coordination likely occurring via side-chain donor groups (*e.g.,* amino or carboxylate groups), as implied from the presence of peptide-platinum adducts and changes in peptide fragmentation patterns. UV-Vis spectroscopy and CD analyses revealed changes in complex absorbance and peptide secondary structure, respectively, consistent with disruption of the β-sheet transitions characteristic of amyloid fibrils. Importantly, NMR spectroscopy supported a model where the platinum center undergoes ligand exchange to coordinate the peptide, stabilizing less toxic oligomeric forms. Microscopy studies further validated these findings by showing that **Pt58**-treated peptide samples form amorphous, non-fibrillar aggregates rather than the classical amyloid fibers. Although **Pt58** (and, to a lesser degree, **Pt57**) can suppress peptide aggregation, cell viability assays demonstrated that these platinum complexes do not rescue neuronal cells from the toxicity induced by amyloid peptides. This limits their immediate utilization as neuroprotective drugs, even though they are strong aggregation inhibitors. In summary, these findings broaden the application scope of metal-glycoconjugates beyond cancer, into neurodegeneration, and provide a basis for developing refined metal-based inhibitors of pathological protein aggregation.

## 6. Conclusions

The altered glucose metabolism observed in tumor cells presents a promising opportunity for the development of more selective anticancer therapies and diagnostic techniques. In this context, “Trojan Horse”-type metallodrugs conjugated to carbohydrate moieties may offer a dual advantage by combining the cytotoxic properties of metal-based therapeutics with the enhanced tumor selectivity potentially achieved through the use of glucose-like or glycomimetic scaffolds designed to exploit the overexpression of glucose transporters (GLUTs) commonly found in cancer cells. Such a strategy holds significant promise since it is expected to facilitate targeted intracellular uptake and delivery of the metal-containing cytotoxic agents, thereby improving therapeutic efficacy while minimizing off-target side effects.

The topic of metal-glycoconjugation for potential medicinal applications has been previously reviewed (see, for example, references [21,22]). However, the development of new synthetic strategies and the improvement of existing synthetic routes to the generation of carbohydrate scaffolds and their metal derivatives, as well as the introduction of innovative biomolecular analytical tools, have significantly advanced research efforts into the design of bioactive glyco-functionalized metal complexes in recent years. Therefore, on account of the aforementioned considerations, we have provided here as comprehensive an overview as possible of the advances made more recently in the field of metal-glycoconjugates as (targeted) chemotherapeutic and tumor-imaging agents. Considering the breadth of the subject, we focused on small-molecule systems containing various metal centers for which significant biological studies were reported, while we decided to exclude topics such as cyclodextrin derivatives, nanoparticle-based platforms, polymeric carriers, and macromolecular assemblies.

When we first reviewed the topic 10 years ago, we then commented on the fact that several metal-glycoconjugates were reported, but their biological evaluation was mostly limited to routine cytotoxicity studies, with very little insight into the connection between bioactivity, cell uptake and capability to target GLUTs [21]. On the contrary, we have now observed a major change of course with the inclusion of detailed mechanistic studies either supporting the rationale of the various research works or providing new knowledge for further developments.

Therefore, upon reviewing the many original research papers discussed here, we believe that the following points would nicely summarize the major advances achieved in the field during the last ten years.

- Although platinum scaffolds (especially Pt(II)-based) still account for the largest class of metal-glycoconjugates, other metal centers are being explored more and more often to exploit alternative transport pathways and mechanisms of action. Interestingly, along with ruthenium complexes, other metal ions such as gold, zinc, iron and lanthanides are now gaining ground in the rush to metal-glycoconjugation.
- Glucose and glucose-like moieties are still the most widely employed scaffolds for metal-glycoconjugation. However, it was proven in many cases that more favorable outcomes may be obtained with other monosaccharides, in particular galactose and mannose.
- The exploitation of GLUTs overexpressed in cancer cells to achieve selective tumor-targeting is still the first choice, driving the overall design of metal complexes functionalized with carbohydrates. However, it has been clearly demonstrated that metal-glycoconjugates may turn out to be biologically active without necessarily being transported and/or taken up by cells through GLUTs (and the other way around).
- As previously pointed out, detailed mechanistic studies have unveiled a number of unexpected targets for different metal-glycoconjugates (for instance, SGLTs or GLUTs other than the expected GLUT1).
- Contrary to the past, when the large majority of metal-glycoconjugates had been developed only as potential anticancer agents, such compounds have been designed also for different bio-applications. In particular, a major push to the generation of lanthanide-based tumor imaging probes has been observed, as well as the development of glyco-functionalized small metal complexes for non-oncological applications (*e.g.,* antivirals and antimicrobials).

Glycoconjugation of metal complexes has undoubtedly strong potential in an attempt to develop new and selective metallodrugs for a variety of medicinal applications. On the other hand, too little is known yet about how glyco-mimetics are metabolized, and more in-depth mechanistic insights are needed. In this context, we believe that the present comprehensive review of the advances in the field recently achieved will inspire scientists to drive this kind of research forward.

## Data Availability

Not applicable.

## Abbreviations

The following abbreviations are used in this manuscript:

2-DG	2-deoxy-d-glucose
2-FDG	2-fluoro-2-deoxy-d-glucose
2-NBDG	(2-[*N*-(7-nitrobenz-2-oxa-1,3-diazol-4-yl)amino]-2-deoxy-d-glucose)
acetyl-CoA	acetyl-coenzyme A
ADEPT	antibody-directed enzyme prodrug therapy
ATP	adenosine-5’-triphosphate
Cp	cyclopentadienyl
DTPA	diethylenetriaminepentaacetic acid
EDG	4,6-*O*-ethylidene-α-d-glucose
FADH_2_	flavin adenine dinucleotide
GLUT	glucose transporter
HIF-1a	hypoxia-inducible factor 1 alpha
MMP	matrix metalloproteinase
MRI	magnetic resonance imaging
MTD	maximum tolerated doses
NADH	nicotinamide adenine dinucleotide
NADPH	nicotinamide adenine dinucleotide phosphate
NHC	*N*-heterocyclic carbene
OCT2	organic cation transporter 2
OXPHOS	mitochondrial oxidative phosphorylation
PACT	photoactivated chemotherapy
PARP-1	poly(ADP-ribose) polymerase 1
PDK1	pyruvate dehydrogenase kinase 1
PDT	photodynamic therapy
PET	positron emission tomography
RNase	ribonuclease
ROS	reactive oxygen species
SGLT	sodium-dependent transporters
SOD	superoxide dismutase
SPECT	single photon emission computed tomography
TBP	trigonal bipyramidal
TRES	time-resolved emission spectroscopy
TrxR	thioredoxin reductase
TUNEL	terminal deoxynucleotidyl transferase dUTP nick-end labeling
